# Reactive Oxygen Species: A Double-Edged Sword in the Modulation of Cancer Signaling Pathway Dynamics

**DOI:** 10.3390/cells14151207

**Published:** 2025-08-06

**Authors:** Manisha Nigam, Bajrang Punia, Deen Bandhu Dimri, Abhay Prakash Mishra, Andrei-Flavius Radu, Gabriela Bungau

**Affiliations:** 1 Department of Biochemistry, Hemvati Nandan Bahuguna Garhwal University, Srinagar Garhwal 246174, Uttarakhand, India; m.nigam@hnbgu.ac.in (M.N.); pooniabajrang56@gmail.com (B.P.); deenbandhudimri20@gmail.com (D.B.D.); 2 Cosmetics and Natural Products Research Center (CosNat), Faculty of Pharmaceutical Sciences, Naresuan University, Phitsanulok 65000, Thailand; 3 Doctoral School of Biomedical Sciences, University of Oradea, 410087 Oradea, Romania; gbungau@uoradea.ro; 4 Department of Psycho-Neurosciences and Recovery, Faculty of Medicine and Pharmacy, University of Oradea, 410073 Oradea, Romania

**Keywords:** cancer, ROS, oxidative stress mediators, oncogenic signaling circuits, antioxidants, programmed cell death

## Abstract

Reactive oxygen species (ROS) are often seen solely as harmful byproducts of oxidative metabolism, yet evidence reveals their paradoxical roles in both promoting and inhibiting cancer progression. Despite advances, precise context-dependent mechanisms by which ROS modulate oncogenic signaling, therapeutic response, and tumor microenvironment dynamics remain unclear. Specifically, the spatial and temporal aspects of ROS regulation (i.e., the distinct effects of mitochondrial versus cytosolic ROS on the PI3K/Akt and NF-κB pathways, and the differential cellular outcomes driven by acute versus chronic ROS exposure) have been underexplored. Additionally, the specific contributions of ROS-generating enzymes, like NOX isoforms and xanthine oxidase, to tumor microenvironment remodeling and immune modulation remain poorly understood. This review synthesizes current findings with a focus on these critical gaps, offering novel mechanistic insights into the dualistic nature of ROS in cancer biology. By systematically integrating data on ROS source-specific functions and redox-sensitive signaling pathways, the complex interplay between ROS concentration, localization, and persistence is elucidated, revealing how these factors dictate the paradoxical support of tumor progression or induction of cancer cell death. Particular attention is given to antioxidant mechanisms, including NRF2-mediated responses, that may undermine the efficacy of ROS-targeted therapies. Recent breakthroughs in redox biosensors (i.e., redox-sensitive fluorescent proteins, HyPer variants, and peroxiredoxin–FRET constructs) enable precise, real-time ROS imaging across subcellular compartments. Translational advances, including redox-modulating drugs and synthetic lethality strategies targeting glutathione or NADPH dependencies, further highlight actionable vulnerabilities. This refined understanding advances the field by highlighting context-specific vulnerabilities in tumor redox biology and guiding more precise therapeutic strategies. Continued research on redox-regulated signaling and its interplay with inflammation and therapy resistance is essential to unravel ROS dynamics in tumors and develop targeted, context-specific interventions harnessing their dual roles.

## 1. Introduction

Cancer is characterized by uncontrolled cell proliferation, evading the body’s inherent regulatory mechanisms. Classification is traditionally based on the tissue of origin, but increasingly, molecular characteristics are considered, leading to various types with differences in their management [1,2]. Globally, cancer is a leading cause of death, posing a significant challenge to increasing life expectancy. In 2020, there were approximately 19.3 million new cancer cases and nearly 10 million deaths, underscoring the need for an optimized understanding of pathophysiology and therapeutic strategies across various cancer types [3].

Within the realm of cancer progression, inflammatory processes can contribute through diverse mechanisms. These encompass the stimulation of cell proliferation, the initiation of immune suppression, the facilitation of tissue remodeling, and the induction of deoxyribonucleic acid (DNA) damage [4]. Moreover, tumor-infiltrating myeloid cells not only drive carcinogenesis via cancer-related inflammatory processes but also significantly impact tumor development, invasion, and metastasis within the medical context [5].

Reactive oxygen species (ROS) serve as cell signaling molecules and play a role in various human diseases and aging processes. In the intricate landscape of cancer biology, a pivotal role is attributed to ROS, dynamic molecules generated during oxidative metabolism. Aging and cancer share biological links, with processes such as DNA damage responses, oxidative stress, metabolic changes, and cellular senescence contributing to both phenomena [6].

ROS encompass a plethora of reactive entities and the free radicals generated via molecular oxygen (O_2_) [7]. They usually originate through the consumption of oxygen molecules, cellular disintegration, and the partial combustion of the molecules of oxygen [8]. The hydroxyl radical (•OH) and superoxide anion (O_2_^•−^) are the major free radical ROS, while hydrogen peroxide (H_2_O_2_) is the main non-radical ROS [7,9]. The mitochondrial electron transport chain (ETC), as a primary intracellular producer of ROS, is followed by a variety of enzymatic sources, including xanthine oxidase (XO), nicotinamide adenine dinucleotide phosphate (NADPH) oxidase (NOX), lipoxygenase, and cytochrome P450. Ionizing radiation (e.g., gamma rays, X-rays, and ultraviolet radiation), contaminants, xenobiotics, and chemotherapeutic drugs are only a few extracellular factors that significantly contribute to an upsurge in ROS levels [10,11]. The biological function of ROS involves cell signaling and cellular homeostasis, which makes them vital for physiological processes.

The pathogenic and physiological roles of ROS result in an enormous paradox in terms of their studies. Researchers widely acknowledge the dual role of ROS in cancer progression, as they have context-dependent effects on tumor growth, metastasis, and apoptosis. ROS exhibit contrasting impacts based on their distribution, concentration, and duration in specific cellular structures. Their roles include ROS-dependent malignant transformation and oxidative stress-induced cell death [12]. At moderate concentrations, ROS trigger a signaling cascade crucial for cancer cell survival. This cascade involves the activation of c-Jun N-terminal kinase (JNK), mitogen-activated protein kinase/extracellular signal-regulated protein kinases 1/2 (MAPK/ERK1/2), phosphoinositide-3-kinase/protein kinase B (PI3K/Akt), and p38. Consequently, these pathways activate matrix metalloproteinases (MMPs), nuclear factor kappa-light-chain-enhancer of activated B cells (NF-κB), and vascular endothelial growth factor (VEGF). Conversely, at higher concentrations, ROS induce apoptosis in cancer cells [13].

Throughout different cancer stages, abnormal ROS levels play paradoxical roles in cell fate. Physiological ROS concentration equilibrium is vital for normal cell survival. Aberrant ROS accumulation promotes cell proliferation, initiating malignant transformation. Conversely, excessive ROS levels induce cell death by damaging cellular components. Strategies involving both scavenging elevated ROS to prevent early neoplasia and promoting ROS production to selectively eliminate cancer cells show promise despite their complexity [12,13].

The implications of ROS in cancer biology have been documented in numerous comprehensive reviews. Several studies have examined ROS-mediated signaling pathways in cancer, highlighting their dual pro-tumorigenic and anti-tumorigenic effects [13,14,15]. Perillo et al. (2020), for instance, discussed ROS as potential “Trojan horses” in cancer therapy, emphasizing their involvement in programmed cell death mechanisms [16]. Other reviews have addressed the paradoxical nature of ROS, illustrating how elevated ROS levels contribute to apoptosis, whereas lower levels promote cell survival, proliferation, and metastasis [17,18]. While the involvement of ROS in cancer biology has been extensively reviewed, substantial gaps persist in our mechanistic understanding, particularly concerning context-specific effects and spatiotemporal regulation. The present review summarizes and critically analyzes the current data on the mechanisms underlying compartment-specific ROS signaling (mitochondrial versus cytosolic), temporal dynamics (acute versus chronic exposure), and the distinct contributions of specific ROS-generating enzymes to tumor microenvironment remodeling. Furthermore, this work uniquely integrates source-specific ROS functions with redox-sensitive pathway modulation, offering novel therapeutic insights that bridge fundamental mechanisms with clinical applications.

Our review delivers a comprehensive and critical analysis of the dualistic role of ROS in cancer, highlighting both their tumor-suppressive and pro-oncogenic functions. Furthermore, it seeks to provide a comprehensive overview of current insights into the biological activities of ROS, their intricate connections with cancer pathophysiology, and their influence on therapeutic interventions and the modulation of key signaling pathways. This work makes a significant scientific contribution by offering an exhaustive evaluation of the molecular signaling cascades driven by ROS in cancer, with particular emphasis on mechanisms underlying tumor progression, cellular proliferation, and metastasis. Through this approach, it aims to substantially advance the existing body of knowledge, offering valuable perspectives that can inform future research and therapeutic development.

## 2. Methodology of Research

This narrative review systematically filters, selects, and critically analyzes the scientific literature addressing the dualistic functions of ROS across various cancer signaling pathways. It explores their influence on multiple cancer-related mechanisms, including tumorigenesis, modulation of inflammatory responses, metastasis, and angiogenesis, as well as programmed cell death processes, such as apoptosis and autophagy. The objective is to provide an up-to-date synthesis of current knowledge in this rapidly evolving field, underscoring the necessity for ongoing research and continuous refinement of our understanding.

The search terms used in databases with wide coverage in the medical field (e.g., Web of Science, PubMed, SciFinder, Scopus, and Google Scholar) included a series of keywords relevant to the subject matter linked through Boolean operators and thus constituting the search algorithm (i.e., “ROS”, “cancer”, “angiogenesis”, “tumorigenesis”, “apoptosis”, “ROS AND cancer”, “ROS AND cancer AND dual role”, “ROS AND inflammation AND cancer”, and “(ROS OR antioxidants) AND cancer”).

The algorithm filters targeted publications written only in English and containing predominantly article- or book-type papers. However, to critically comment on the reported data and assess and compare the most intriguing studies, several valuable papers written in the first decade of the 2000s were described. Finally, a total of 374 scientific literature references were selected, evaluated, and cited to validate the information in the present narrative review.

## 3. ROS: Dusk and Dawn

ROS derived from both endogenous and exogenous sources are constantly produced and removed from cells via the antioxidant system. The mitochondrial ETC is a primary endogenous source of ROS in cells. In the ETC, single electron reduction of O_2_ is performed by ubiquinone oxidoreductase (complex I) and ubiquinol–cytochrome c oxidoreductase (complex III), and these are the major sites for the production of O_2_^•−^, which is further transported to the cytosol by diffusion via voltage-dependent anion channels [19]. Next to the ROS-generating endogenous source is NOX, which is mostly found in the plasma membrane of white blood cells (neutrophils). The NOX family consists of seven isoforms of enzymes, including NOX1-5 and dual oxidase 1–2 (DUOX1, DUOX2), which have the sole function of producing ROS [20]. O_2_^•−^ is mainly produced by NOX1-3, and H_2_O_2_ is mainly produced by NOX4, DUOX1, and DUOX2 [21].

The conversion of purine nucleotides to uric acid is an essential contributor to producing ROS. XO catalyzes this reaction and accounts for the production of both O_2_^•−^ and H_2_O_2_ [22]. It is known that O_2_^•−^ is also produced by XO in the peroxisome [23]. The family of non-heme iron-containing enzymes known as lipoxygenases (LOXs) catalyzes the deoxygenation of polyunsaturated fatty acids in lipids. In order to create hydroperoxide derivatives from polyunsaturated fatty acids and hence increase ROS, the LOX family plays a crucial role [24]. Similarly, during the formation of steroids, cytochrome P450 plays a crucial role in breaking down oxygen molecules. During the breakdown of oxygen molecules, ROS are generated [25]. It has also been reported that ROS are generated during protein folding in the endoplasmic reticulum, as well as during the activity of prostaglandin-producing cyclooxygenases (COXs) [26,27].

In addition to endogenous sources, cellular ROS levels can also increase following exposure to exogenous sources. Ionizing radiation has a hazardous effect on the skin; it energizes the electrons of oxygen and continuously produces ROS [28]. Some chemotherapeutic drugs, like doxorubicin and cisplatin, show selective killing of cancer cells by producing mitochondrial ROS [29]. Chemicals in cigarette smoke, including carcinogens and mutagens (i.e., benzo[a]pyrene, formaldehyde, nitrosamines, and arsenic), can generate ROS either directly or indirectly, leading to oxidative stress and cellular damage [30,31]. Numerous contaminants, including heavy metals like As, Cd, Cr, Hg, and Pb, have been identified as direct causes of ROS. Intake and accumulation of the air contaminants in the respiratory tract can rise and prolong redox homeostasis, which indirectly produces ROS [32,33].

Although smoking is a well-established risk factor for oral precancerous lesions, certain findings revealed a higher incidence among non-smokers, suggesting a potentially controversial and multifactorial etiology. This paradox may involve various contributors such as genetic predisposition, environmental exposure, exogenous ROS sources, and other lifestyle factors [34].

ROS in excessive amounts should be cleared from cells because it is linked to numerous diseases. Across many subcellular compartments, the antioxidant system maintains the equilibrium between generation and clearance. There are two types of antioxidant systems: enzymatic systems, like the glutathione peroxidase (GPx) system, the peroxiredoxin (Prx) system, superoxide dismutase (SOD), catalase, etc., and non-enzymatic systems, like α-tocopherol, ascorbic acid, lipoic acid, etc. [35].

**Figure 1:**
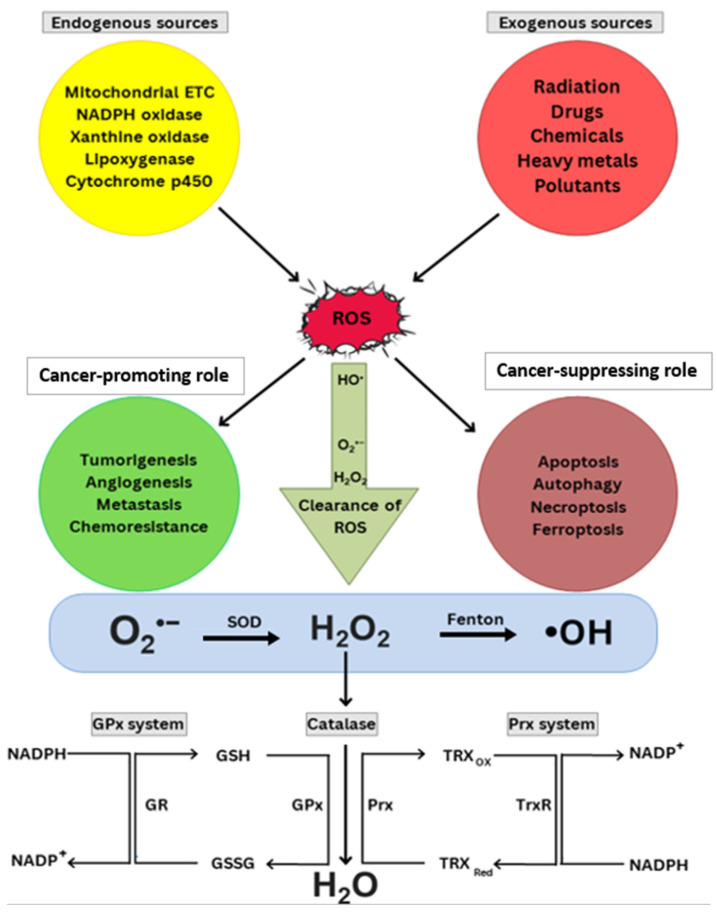
ROS sources and transformation. The main endogenous and exogenous sources of ROS molecules are depicted in this image, along with the antioxidant mechanisms that catalyze the reduction of various ROS molecules. It also shows the Fenton reaction that produces HO•. NADPH, nicotinamide adenine dinucleotide phosphate (reduced form); NADP+, nicotinamide adenine dinucleotide phosphate (oxidized form); GR, glutathione reductase; GSH, reduced glutathione; GPx, glutathione peroxidase; GSSG, oxidized glutathione; TRX ox, oxidized thioredoxin; Prx, peroxiredoxin; TRX red, reduced thioredoxin; SOD, superoxide dismutase; HO•, hydroxyl radical; O_2_^•−^, superoxide ion; H_2_O_2_, hydrogen peroxide.

O_2_^•−^ dismutation into H_2_O_2_ is catalyzed by SOD and, subsequently, further reduction of H_2_O_2_ is performed via Prx, GPx, and the catalase enzyme. SODs can be categorized into three types: Cu/ZnSOD (SOD1) in the cytoplasm, MnSOD (SOD2) in the mitochondria, and extracellular Cu/ZnSOD (SOD3). Each isoform contains a catalytic metal ion (either copper or manganese) as an essential cofactor in its active site [36]. In the Prx system, TRX is subsequently reduced and oxidized because it works as an intermediary that transfers electrons between NADPH and Prx. TRX takes electrons from NADPH and is reduced; then, it transfers electrons to Prx and is oxidized again. Then, Prx donates its electrons to H_2_O_2_ and is oxidized again; this cycle repeats again and again until H_2_O_2_ is converted to H_2_O. In the GPx system, glutathione acts as an intermediate electron donor between NADPH and GPx, as illustrated in Figure 1. Similar to the Prx system, it converts H_2_O_2_ to H_2_O in the final step [37].

## 4. ROS in Cancer Promotion

**Figure 2:**
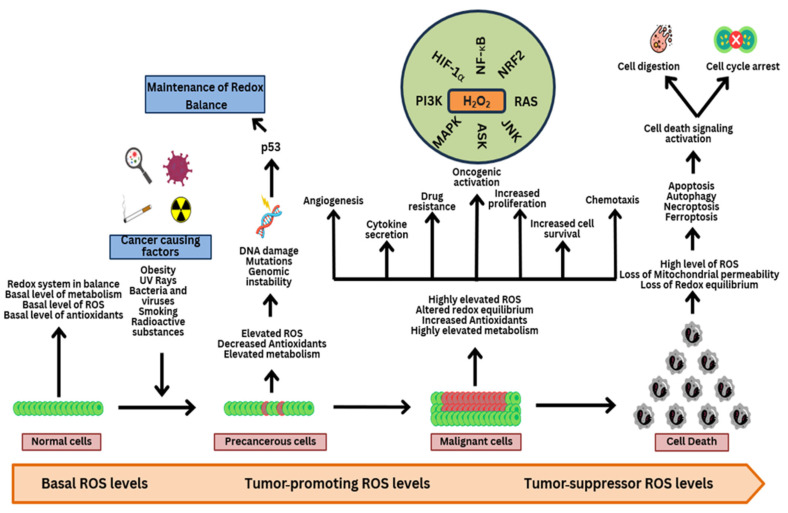
Implications of ROS in cancer. Under homeostatic circumstances, the redox system is in balance in normal cells (green cell), with lower intracellular ROS levels. Factors that boost ROS levels, including obesity, smoking, and radiation exposure, cause DNA damage, genomic instability, and the emergence of cancer-driving mutations in precancerous lesions (red cells). When exposed to factors that increase the risk of developing cancer, tumor suppressor proteins like p53 become active and trigger the transcription of genes related to antioxidant defense. As opposed to this, when repair mechanisms are insufficient, ROS and/or antioxidant levels increase, resulting in an altered redox balance that favors the activation of intracellular oncogenic signaling pathways and maintains the functionality of cellular components in cancer cells (red cells). These routes are often linked to enhanced cytokine release, drug resistance, metastasis, cell survival, and proliferation. Eventually, a breakdown in redox equilibrium brought on by an excessive rise in ROS caused by an overstrained antioxidant system results in apoptosis and necrosis. At this stage, p53 activation brought on by significant cellular damage may result in cell cycle arrest, senescence, and cell death (grey cells), possibly indicating that the response of a variable ROS depends on the tumor’s p53 status and/or oncogenic background. HIF-1α, hypoxia-inducible factor 1-alpha; NF-κB, nuclear factor kappa-light-chain-enhancer of activated B cells; NRF2, nuclear factor erythroid 2-related factor 2; RAS, renin–angiotensin system; JNK, c-Jun N-terminal kinase; ASK, apoptosis signal-regulating kinase; MAPK, mitogen-activated protein kinase; PI3K, phosphoinositide 3-kinase; H_2_O_2_, hydrogen peroxide.

Oxidative stress disrupts cellular lipids, proteins, and DNA, which consequently impacts the redox equilibrium of cells. Long-term metabolic defects result in high ROS levels. In addition to being detrimental, ROS are also crucial for the signaling pathways implicated in cancer. The development of numerous malignancies, such as leukemia, glioma, hepatoma, and melanoma, including cancers of the pancreas, breast, lungs, prostate, bladder, and colon [38], is strongly influenced by signaling pathways that are elevated by ROS, as shown in Figure 2. Growth factors, cell–cell adhesion, and inflammation are certain important features of cancer that have been linked in one way or another to cancer initiation, malignant transformation, and chemotherapy resistance. ROS may impact these factors in a cell. The documented roles of ROS in the promotion of cancer will be addressed in this section, and Table 1 includes some of the factors that may trigger cancer by activating ROS-mediated cell signaling pathways.

### 4.1. ROS and Tumorigenesis

Tumorigenesis is the term used to describe how a tumor initially develops in the body. Tumor development is a result of genetic instability and the induction of several signaling pathways. Oncogene activation and suppressor gene inactivation are intimately correlated with DNA damage. The level and length of ROS exposure determine the degree of cellular protein, lipid, and eventual DNA damage [72]. It is apparent that because mitochondrial DNA is exposed to ROS more frequently than nuclear DNA, it is more susceptible to DNA damage. The inactivation of the tumor suppressor protein, phosphatase and tensin homolog (PTEN), which consequently hyperactivates PI3K/Akt (phosphoinositide 3-kinase) oncogenic signaling, is another example of the manner in which ROS and cancer are related [73].

Until recently, it was unclear how ROS affected the development of cancer, but recent research and studies have demonstrated these mechanisms. By regulating redox-sensitive transcription factors, cell membrane lipids, cell cycle regulators, kinases, and phosphatases, ROS have an impact on a few signaling pathways. Most ROS oxidize cysteine residues containing thiol groups to form disulfide bonds, thereby altering proteins in a way that can either enhance or deactivate how they function. The NF-κB, mitogen-activated protein kinases (MAPKs), nuclear factor erythroid 2-related factor 2 (NRF2), hypoxia-inducible factor (HIF-1α), PI3K, and p53 are a few of the pathways that are regulated, which are discussed in the next section [74,75,76].

#### 4.1.1. NF-κB Regulation

NF-κB plays a vital function in regulating DNA transcription, manufacturing cytokines, cell survival, and the immune system’s response to infection. The IκB kinase (IKK) complex, which comprises the two kinases IKKα and IKKβ, as well as the NF-κB essential modulator (NEMO), a regulatory subunit, is a crucial modulator of all inducible NF-κB signaling pathways. NF-κB dysregulation has been implicated in autoimmune, inflammatory, and cancerous pathologies. NF-κB can activate the genes for ROS-producing enzymes, such as cyclooxygenase-2 (COX2), NADPH oxidase 2 (NOX2), inducible nitric oxide synthase (iNOS), XO, and cytochrome P450, during the inflammatory phase [77]. The proteins c-Rel, p65/RelA, RelB, p50/p105 (NF-κB 1), and p52/p100 (NF-κB2) comprise the NF-κB transcription factor family. The activated dimers and homodimers of these proteins cause the targeted genes to be transcribed. Depending on the form of cell or subcellular location of the ROS, their interaction with the NF-κB pathway may either promote or block NF-κB-mediated transcription [27].

The IκB proteins play a major role in controlling NF-κB activity. IκBα, IκBβ, and IκBε are three of these proteins that are regarded as “typical” IκBs. Reactive oxygen species (ROS) promote the activation of the NF-κB pathway in part through the cAMP-dependent protein kinase A (PKA)-mediated phosphorylation of the p65 subunit. Specifically, phosphorylation at Ser276 enhances p65’s affinity for the transcriptional coactivators CBP/p300, a modification essential for robust transcription of NF-κB target genes [78,79].

ROS modulate NF-κB both positively and negatively through redox-sensitive modifications: they can enhance IKKβ activity via Cys oxidation and NEMO dimerization while also inhibiting the pathway through glutathionylation of IκBα, S-nitrosylation of p65, or oxidation of p50 [78,80,81,82].

Simultaneously, S-glutathionylation of IκBα, a reversible ROS-triggered post-translational modification (PTM), can prevent its degradation, thereby attenuating NF-κB activation in a compartment-specific manner [83]. Likewise, S-nitrosylation of the p65 subunit on Cys38 inhibits its DNA-binding activity, acting as a redox checkpoint in transcriptional output [84].

Emerging evidence shows that O-linked β-N-acetylglucosamine glycosylation (O-GlcNAcylation) of p65 on Thr352, a nutrient/redox-sensitive modification, competes with phosphorylation and stabilizes nuclear NF-Κb, prolonging transcription of anti-apoptotic genes [85].

Subcellular ROS localization, such as NOX-derived ROS at the membrane or mitochondrial ROS in the cytoplasm, distinctly modulates NF-κB signaling [86]. In the nucleus, oxidative stress modifies chromatin-bound p65 through acetylation and redox-sensitive histone crosstalk [87], while endoplasmic reticulum stress-derived ROS synergize with inositol-requiring enzyme 1-TRAF2 to activate NF-κB independently of canonical IKK pathways [88]. This layered control allows NF-κB to fine-tune gene expression linked to inflammation, angiogenesis, and proliferation [89], redox homeostasis [90], and drug resistance mechanisms (*ABCC1* and *ABCB1*) [91].

The NF-κB pathway is activated in cancer and has been linked to resistance to apoptosis because the anti-apoptotic proteins Bcl-2 and Bcl-XL are negatively regulated by the NF-κB pathway. By altering the gene expression related to cell proliferation (cyclin D1 and c-Myc), angiogenesis (VEGF), and various pro-inflammatory cytokines (IL-1 and IL-6) linked to cancer, it increases malignancy. Additionally, it modifies genes related to resistance to drugs, detoxification, and enzymatic antioxidants (CAT, SOD, GPX1, and TRX) [27,92,93].

While NF-κB is well established as a redox-sensitive transcription factor promoting tumor survival and inflammation, its activation is not uniformly pro-tumorigenic. In some contexts, ROS-mediated NF-κB signaling may sensitize cells to apoptosis or immune responses. The pathway’s dual role depends on specific ROS species, concentration, and cell type, raising challenges for selectively targeting NF-κB in cancer therapy.

#### 4.1.2. PI3K/Akt Regulation

**Figure 3:**
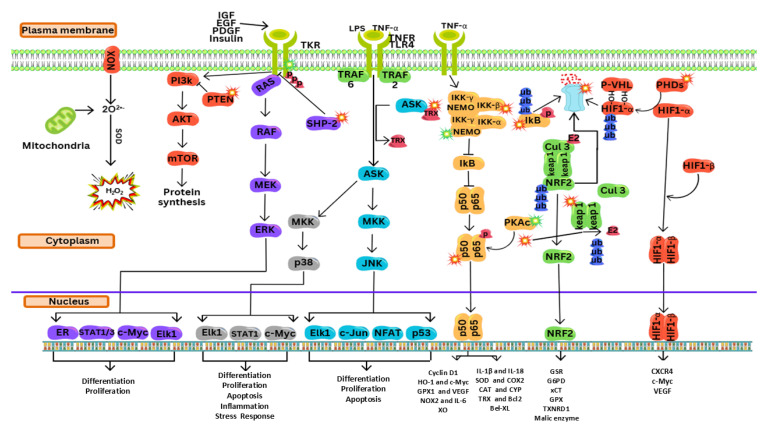
ROS-mediated signaling channels. Proteins implicated in cancer-related signaling pathways can be inactivated by ROS (red burst) or activated (green burst) by ROS. ROS stimulate the NF-κB pathway, favoring dimerization of IKK/NEMO and inducing phosphorylation of NF-kB inhibitors (IBs), which are later destroyed by the proteasome. ROS also cause the translocation of NF-κB to the nucleus, causing or inhibiting the transcription of genes involved in cell survival, proliferation, and ROS. Once Keap1 is oxidized, NRF2 is activated, which prevents Keap1 from binding to NRF2, causes NRF2 to relocate to the nucleus, and triggers the transcription of genes that respond to antioxidants. Prolyl-hydroxylases (PDHs), which are essential for joining the p-VHL protein with HIF-1, are also more likely to be oxidized by ROS, leading to their ubiquitination and destruction in the proteasome. In the presence of ROS, TRX oxidizes and separates from ASK. This causes the phosphorylation of MKK, which, in turn, phosphorylates JNK or p38. This facilitates the transcription of genes involved in cell division and proliferation, apoptosis, inflammation, and stress response. As a result of ROS inhibiting SHP-2 phosphatase, TRK receptors continue to be phosphorylated and activate cell signaling, including the RAS-RAF-MEK-ERK pathway, which, in turn, stimulates the transcription of genes that control cell proliferation and differentiation. Lastly, ROS oxidize and inactivate PTEN phosphatase, which encourages the activation of the PI3K/AKT pathway. IGF, insulin-like growth factor; EGF, epidermal growth factor; PDGF, platelet-derived growth factor; TKR, tyrosine kinase receptor; NOX, NADPH oxidase; SOD, superoxide dismutase; H_2_O_2_, hydrogen peroxide; ER, endoplasmic reticulum; STAT1/3, signal transducer and activator of transcription 1/3; c-Myc, cellular Myc oncogene; Elk1, Ets-like protein-1; PI3k, phosphoinositide 3-kinase; AKT, protein kinase B; mTOR, mammalian target of rapamycin; PTEN, phosphatase and tensin homolog; RAS, renin–angiotensin system; RAF, rapidly accelerated fibrosarcoma; SHP-2, Src homology 2 domain-containing phosphatase-2; MEK, mitogen-activated protein kinase; ERK, extracellular signal-regulated kinase; MKK, MAP kinase; TRAF6, TNF receptor-associated factor 6; LPS, lipopolysaccharide; TNF-α, tumor necrosis factor-alpha; TNFR, TNF receptor; TLR4, Toll-like receptor 4; TRAF2, TNF receptor-associated factor 2; ASK, apoptosis signal-regulating kinase; JNK, c-Jun N-terminal kinase; IKK-β, IκB kinase-beta; IKK-α, IκB kinase-alpha; NEMO, NF-kappa-B essential modulator; IkB, inhibitor of kappa B; p50, nuclear factor kappa B subunit 1; p65, nuclear factor kappa B subunit 3; PKAc, protein kinase A catalytic subunit; keap 1, Kelch-like ECH-associated protein 1; Cul 3, Cullin-3; NRF2, nuclear factor erythroid 2-related factor 2; P-VHL, von Hippel–Lindau tumor suppressor protein; HIF1-α, hypoxia-inducible factor 1-alpha; PHDs, prolyl hydroxylases; HIF1-β, hypoxia-inducible factor 1-beta; NFAT, nuclear factor of activated T cells; p53, tumor protein p53; Cyclin D1, cyclin D1; HO-1, heme oxygenase-1; GPX1, glutathione peroxidase 1; VEGF, vascular endothelial growth factor; NOX2, NADPH oxidase 2; IL-6, interleukin-6; ΧO, choline oxidase; IL-1ß, interleukin-1 beta; iNOS, inducible nitric oxide synthase; COX2, cyclooxygenase-2; CAT, catalase; CYP, cytochrome P450; TRX, thioredoxin; Bcl-2, B-cell lymphoma 2; Bcl-XL, B-cell lymphoma-extra-large; GSR, glutathione reductase; G6PD, glucose-6-phosphate dehydrogenase; xCT, cystine/glutamate transporter; GCL, glutamate–cysteine ligase; GPX, glutathione peroxidase; PRDX, peroxiredoxin; TXNRD1, thioredoxin reductase 1; CXCR4, C-X-C chemokine receptor type 4.

As illustrated in Figure 3, carcinogenesis has a direct relationship with PI3K/Akt activation, one of the most frequently activated signal transduction pathways in numerous human malignancies. Extracellular growth factors, including platelet-derived growth factor (PDGF) and epidermal growth factor (EGF), and insulin also activate Class I PI3K and the associated downstream mediator Akt/PKB [94]. This phosphorylation signaling cascade then activates mTOR kinase and RAS/MAPK (a MAPK pathway comprising several key signaling components like RAS). Lipid phosphatases act as negative regulators of PI3K signaling, with the phosphatase and tensin homolog (PTEN) protein being the most prominent member, frequently mutated or deleted across various human cancers, leading to constitutive activation of the PI3K/Akt pathway [94,95].

It is widely recognized that growth factor receptor signaling, which essentially regulates protein phosphorylation, depends on the generation of H_2_O_2_. Due to the acidic cysteine residue in their active site, protein tyrosine phosphatases (PTPs) have been suggested as targets of oxidation by H_2_O_2_ in cells stimulated by growth factor signaling [96,97,98].

ROS, particularly H_2_O_2_, exert nuanced control over PI3K/Akt signaling via redox-regulated PTMs [99]. Akt activation involves canonical phosphorylation at Thr-308 by 3-phosphoinositide-dependent protein kinase-1 and Ser-473 by mTORC2, but ROS exposure accelerates and prolongs these events [100,101]. Concurrently, oxidation of cysteine pairs in Akt forms reversible disulfides, shifting its conformation and enhancing kinase activity independently of growth factors [102]. Moreover, O-GlcNAcylation, a dynamic Ser/Thr glycosylation, can occur at or near these phosphorylation sites, competing with phosphorylation and sustaining Akt activation, contributing to chemoresistance and survival signaling in cancer cells [103]. Additionally, sumoylation at Lys276 (K276) enhances Akt activity in a redox-sensitive manner, with small ubiquitin-like modifier (SUMO) E3 ligase protein inhibitor of activated STAT 1 promoting Akt SUMOylation and SUMO-specific protease 1 reversing it; this modification supports proliferation and oncogenic phenotypes linked to ROS [104,105].

Acute ROS exposure drives transient modifications and Akt hyperactivation via PTEN oxidation, while chronic oxidative stress leads to irreversible advanced glycation end-products on Akt lysines, influencing cell fate toward growth arrest or apoptosis. These dynamic and temporally distinct redox PTMs underscore the intricate regulation of Akt in cancer biology [106,107].

PTEN’s central role in antagonizing PI3K/Akt is finely tuned through redox chemistry. The catalytic Cys-124 undergoes reversible oxidation by H_2_O_2_, forming internal disulfides or intermolecular crosslinks, transiently abolishing lipid phosphatase activity. ROS also drive conformational changes that diminish PTEN’s membrane affinity, biasing its activity toward protein substrates and altering downstream signaling [108]. Furthermore, redox-driven aggregation within lipid rafts creates localized zones of low PTEN function, fostering spatial heterogeneity in Akt activation. Distinct ROS sources define this specificity: NOX4-derived H_2_O_2_ primarily oxidizes cytoplasmic PTEN, while mitochondrial superoxide, via SOD2 conversion, targets nuclear PTEN. This compartmentalized redox regulation opens selective strategies to modulate specific PTEN pools in cancer cells [14,97].

Subcellular compartmentalization creates distinct PI3K/Akt signaling subroutines under redox control [109]. Mitochondrial ROS emerge from the electron transport chain and can directly oxidize nuclear or mitochondrial Akt isoforms [110,111,112], especially Akt3, which governs genome integrity and repair mechanisms [113]. These context-specific ROS pools allow cancer cells to fine-tune survival signaling in specific compartments, stimulating proliferation or stress responses based on local ROS concentrations and sources. Targeting individual ROS pools or their generating enzymes, like NOX4 or mitochondrial SOD2, may offer strategies to disrupt tumor-specific signaling while minimizing systemic redox disruption [99].

ROS-dependent modulation of the PI3K/Akt axis shapes both therapy sensitivity and resistance. Oxidative inactivation of PTEN and enhanced Akt signaling can drive resistance to PI3K/Akt inhibitors. Conversely, strategies that re-establish redox balance can restore PTEN function and sensitize tumors [114].

Although ROS-mediated inactivation of PTEN and activation of PI3K/Akt are frequently observed in tumors, emerging evidence indicates that this mechanism may vary depending on the oxidation state of PTEN and its isoforms. Furthermore, ROS involvement in PI3K/Akt signaling is tightly linked to cellular metabolism, making it difficult to generalize therapeutic interventions targeting this axis.

#### 4.1.3. MAPKs Regulation

MAPKs, a form of serine and threonine protein kinases, are engaged in signal transduction pathways that control both beneficial and detrimental cell responses. Intracellular targets phosphorylated upon MAPK activation encompass nuclear pore proteins, membrane transporters, cytoskeletal elements, transcription factors, and other protein kinases involved in the regulation of cellular processes, such as proliferation, migration, differentiation, senescence, or cell death [115,116].

The extracellular signal-regulated kinases (ERK1/2), c-Jun N-terminal kinase (JNK), p38, and growth factors (EGF, PDGF, or insulin) are among the intracellular stressors that might activate MAPKs in response to external triggers. Growth factor signaling is the main source of activation for ERK1/2, while cytokines or intracellular stressors activate JNK and p38 [117]. Oxidative modification of MAPK phosphatases (MKPs), such as MKP-1, by ROS leads to sustained ERK and p38 activation, contributing to tumorigenesis in oxidative microenvironments [118].

Under mitochondrial stress, mitochondrial ROS modulate downstream JNK via direct oxidation of MKK4 at Cys246 and Cys266, leading to enhanced nuclear translocation and transcription factor AP-1 activation [119].

The induction of the RAS/MAPK pathway is intimately correlated with the generation of ROS and is subject to strict regulation at many levels [120]. Thioredoxin (TRX)/Apoptosis Signal-Regulating Kinase 1 (ASK1) are the redox indicators in the MAPK pathway’s cascade. ASK1 activation is further modulated by S-glutathionylation at Cys250, which stabilizes its inactive form. Oxidation-induced disulfide dimerization across Cys30–Cys250 shifts ASK1 toward active homodimers, promoting JNK/p38 signaling [121,122]. O-GlcNAcylation of ASK1 on Thr845 enhances its kinase activity under oxidative stress, linking nutrient/redox status to stress responses [123,124,125].

Inhibition of the kinase activity of ASK1 is characterized by the direct binding of TRX to ASK1. Activation of MAPK is mediated by oxidation of TRX by ROS, which leads to dissociation of TRX and activation of ASK1. This is characterized by phosphorylation of ASK1’s kinase domain on a threonine residue and further JNK and p38 signaling activation [117]. Additionally, it has been shown that ROS can trigger ERK signaling, most likely through upstream epidermal growth factor receptor (EGFR) activation. Considering this, it has been shown that EGFR signaling results in NOX-mediated H_2_O_2_ generation and PI3K activation [126]. It is also known that ROS can maintain EGFR-mediated signaling by causing protease-linked ligand loss from the plasma membrane [127] or by inactivating SHP-2 phosphatase [128]. ROS such as H_2_O_2_ can sulfenylate EGFR at Cys797, augmenting its tyrosine kinase activity and sustaining downstream ERK phosphorylation [129]. ROS also inactivate SHP2 by oxidative phosphorylation of its catalytic Cys459, thereby prolonging EGFR/MAPK signaling [130].

Mutations in the mitochondria that could increase ROS production in the mitochondria have also been linked to MAPK activation. ERK2, or mutant RAS, has been shown to phosphorylate and activate DRP1 in RAS-mutated malignancies, which, in turn, encourages the fission of mitochondria, elevates the mass of mitochondria, and reduces ATP synthesis by mitochondria [131,132].

The RAS/Raf/MEK/ERK1/2 kinase system has been identified as one of the most perturbed mechanisms in cancer because of an excess of growth factors, hormone signaling, or activated oncogenic mutations that lead to inappropriate mitogenic signaling through MAPK, elevated proliferation, and evasion of cell death. Up to 30% of human malignancies have RAS family gene mutations and activation, which stimulates the activation of proteins linked to the motility and intrusiveness of cancer cells, such as Rac, Rho, or PI3K [133]. Thus, while ROS-driven oncogenic pathways, such as PI3K/Akt and MAPK, are widely implicated in tumor initiation and progression, their context-dependent responses, particularly in relation to tumor suppressors like PTEN and p53, highlight a critical need to dissect tumor-type-specific redox adaptations.

#### 4.1.4. NRF2 Regulation

The transcription factor NRF2 controls a large number of cytoprotective genes involved in drug detoxification, autophagy, apoptosis, cell development, metabolism, proteasomes, DNA repair, and antioxidant defense [134,135]. Perhaps the most well-known function of NRF2 is to maintain redox equilibrium by encouraging the expression, synthesis, and redox channeling of glutathione and thiol-based enzymes that are antioxidants. The components of the enzymes glutamate-cysteine ligase (GCL), GPX2, GPX4, GSR, PRDX1/6, TRX1, and thioredoxin reductase 1 (TXNRD1) are among the antioxidant gene targets of NRF2 [135]. NRF2 is a target of two enzymes, glucose-6 phosphate dehydrogenase (G6PD) and malic enzyme 1, that provide NADPH, a cofactor required for GSH or TRX reduction. This suggests that NRF2 either directly or indirectly affects the redox equilibrium [134,136].

Despite the high amounts of drug-detoxifying, cytoprotective, and antioxidant activities that are typically present in cancerous cells, abnormal NRF2 activity in such cells has frequently been seen. Moreover, a component of the Cullin-3 (CUL3)-based ubiquitin ligase complex, KEAP1 (Kelch-like ECH-associated protein 1), controls NRF2 stability and triggers the proteasomal breakdown of NRF2 upon binding. Normal and unstressed conditions result in modest NRF2 levels; nevertheless, NRF2 levels rise quickly when exposed to electrophilic chemicals or ROS that alter the cysteine residues on KEAP1, preventing or weakening its binding to NRF2 or CUL3 [134]. Aside from the nucleus-localized β-TrCP (β-transducing repeat-containing protein), which binds to the phosphorylated form of NRF2 and CUL1, resulting in the ubiquitination of NRF2, the other mechanisms for NRF2 degradation include post-translational SUMOylation, acetylation, or ROS-triggered hypomethylation of the promoter regions of NRF2 [134,135]. A few frequent modifications in NRF2 signaling that have been identified for cancer cells comprise somatic mutations in the KEAP1 or CUL3 genes, epigenetic KEAP1 silencing, accumulation of KEAP1-interacting proteins like p62/SQSTM1, p21, or cysteine modification of an oncometabolite like fumarate [134] (Figure 3). Moreover, stimulation of NRF2 is linked to chemo- and radiation resistance as well as lower recovery chances in head and neck [137], lung [138], ovarian [139], and breast cancers [140].

Conflicting evidence exists regarding the role of NRF2 in cancer. While its activation protects normal cells from oxidative stress, persistent NRF2 signaling has been associated with tumor progression and resistance to chemotherapy. This paradox underscores the dual nature of NRF2, warranting deeper investigation into its time and context-dependent modulation across different tumor microenvironments. Understanding this balance may reveal NRF2 as both a therapeutic target and a resistance mechanism depending on the cancer stage and redox context.

### 4.2. ROS and Metastasis

By conveying signals from the cell surface to central signaling proteins, the production of ROS by cells enriches cell surface receptors with proteins and growth factors that control the motility, invasion, and metastasis of cells. The processes of invasion and metastasis are initiated and carried out by a complex network of molecular signaling channels that control cytoskeletal dynamics, their interaction with the extracellular matrix (ECM), and their movement into the surrounding tissue [141]. A growing amount of research indicates that ROS, particularly H_2_O_2_, actively participate in regulating a variety of processes required for cancer cell growth and dissemination. The physiological importance of epithelial–mesenchymal transition (EMT) in tumor growth and treatment resistance has been extensively shown in studies. The crucial process of EMT, which can transform epithelial cells into mesenchymal cells, is now widely recognized. ROS have an impact on a number of variables, including TGF-β (transforming growth factor-β), TNF-α (tumor necrosis factor-α), HIF-1, 12-O-tetradecanoylphorbol-13-acetate (TPA), and MMPs [142].

#### 4.2.1. Role of TGF-β

Transforming growth factor-β (TGF-β), along with EMT and ROS, is a key player in the progression of cancer. EMT is one of the many biological processes in which TGF-β signaling is engaged [143]. It has been shown that TGF-β and ROS signals interact with one another. While TGF-1 enhances the synthesis of intracellular ROS, p38 MAPK, ERK1/2, phosphorylated Smad 2, α-smooth muscle actin (α-SMA), and fibronectin, it decreases the expression of E-cadherin. Additionally, antioxidants have been reported to remarkably reduce TGF-β1-linked ROS, ERK, p38, and EMT. Moreover, NADPH oxidase inhibitors significantly diminish the ROS generated by TGF-β1. This suggests that ROS plays a critical role in TGF-β1-linked EMT by initially activating MAPK, which, in turn, activates the Smad pathway under ERK’s control [142].

Another regulator of TGF-β1-induced EMT was reported to be the cellular iron-containing protein ferritin, which is composed of 24 peptides of two subunits: ferritin heavy chains (FHCs) and ferritin light chains (FLCs). The levels of FHCs were found to have dropped dramatically in response to TGF-β1-induced EMT, which led to the release of iron from FHCs and an increase in the intracellular pool of labile iron (LIP), in turn promoting the formation of ROS and triggering p38 MAPK. These findings indicate that ROS generation, which is facilitated by increased LIP, is necessary for TGF-β1-induced EMT. Consequently, it is believed that TGF-β plays a significant role in the processes of EMT, suggesting that novel TGF-β inhibitors may be effective against cells that exhibit EMT-like characteristics or cancer stem cells [144].

The interplay between TGF-β and ROS is central to EMT induction, yet the transition from cytostatic to pro-metastatic signaling remains incompletely understood. Variability in ROS thresholds and TGF-β isoform expression complicates therapeutic targeting, suggesting a need for temporally resolved, cell-type-specific profiling.

#### 4.2.2. Role of TNF-α

Several researchers have noted that ROS production is also known to be boosted by inflammatory cytokines, like TNF-α, which are produced by activated monocyte–macrophages. The fact that NF-κB can be activated by several substances, such as radiation, oxidative stress, and the cytokine TNF-α, implies that ROS are an effective regulator of the “master” transcription factor NF-κB. TNF-α may promote EMT of MCF-7 breast cancer cells, according to research. The levels of vimentin and Snail, an essential transcription factor in EMT, are elevated during this process, whereas E-cadherin, the “hallmark” of EMT, is decreased. Moreover, TNF-α-induced EMT is dependent on NF-κB activation since it increases the expression of Snail. Moreover, during TNF-α-induced EMT, the ROS brought on by TNF-α play a small function [145].

Although NF-κB has a minor involvement in TNF-α-induced EMT, H_2_O_2_ alone has been shown to elevate EMT in a manner that is found to be distinct, indicating that TNF-α- and ROS-triggered EMT may be the result of different processes. This can be explained by the fact that the quantity, timing, and compartment of the ROS produced by TNF-α differ from those produced by exogenous H_2_O_2_ treatment during hypoxia or under other circumstances. Moreover, ROS can function as EMT activators, bystanders, or inhibitors in different situations [145]. Studies have demonstrated that the activation of the Rho kinase pathway by ROS-mediated Src kinase led to the development of stress fibers and cell–cell separation. However, due to NF-κB activation and manganese SOD production, the cell–cell separation brought on by oxidative stress was transient. Since H_2_O_2_ therapy alone does not elicit EMT, but TNF-α treatment does, it is believed that NF-κB is a part of a negative feedback mechanism that controls intracellular ROS levels. According to these findings, oxidative stress is essential for cell–cell separation, which is the first step in acquiring EMT features, but it does not offer enough signals for EMT to establish and maintain itself [146]. Even though TNF-α-induced ROS contribute to EMT and metastasis, their role appears to be context-dependent and often transient. Not all ROS generated by TNF-α lead to stable EMT phenotypes, indicating that additional cofactors or sustained redox imbalance may be required for full mesenchymal transformation.

#### 4.2.3. Role of HIF-1α

HIF-1, a transcription factor that consists of the subunits α and β, plays a significant role in how cells respond to hypoxia. Numerous cancers have hypoxia as a common trait; it promotes the growth of local and systemic tumors and lessens the effectiveness of radiotherapy and chemotherapy in eliminating tumors. It has been proposed that the cancer stem cell niche is significantly influenced by hypoxia and HIF-1 activation. Although low oxygen tension may help certain tumor cells die, hypoxia strongly offers a selective pressure that can control tumor development and can start EMT, leading to the aggressiveness of the tumor and resistance to the drug. Cells stabilize HIF-1α in response to hypoxia, which, after dimerization with HIF-1β, translocates into nuclei and causes its target genes to establish a variety of pivotal alterations to effectively combat the drop in oxygen tension. A recent study showed that EMT can boost the invasiveness of several human cancer cells when mild hypoxic conditions exist. A two-phase procedure leads to changes that are hypoxia dependent. For example, early-stage Snail translocation and negative regulation of E-cadherin may lead to EMT phenotypes when ROS block glycogen synthase kinase-3β (GSK-3β). Later, however, persistently activated Wnt/β catenin signaling and VEGF (vascular endothelial growth factor) are involved. These results imply that early redox processes can activate hypoxia-dependent EMT feature development, while HIF-1α-dependent VEGF production sustains rising invasiveness. Taken together, these findings are seen as a vicious cycle of tumor growth and metastasis [147] (Figure 3).

Notably, ROS-mediated stabilization of HIF-1α drives angiogenesis and EMT in hypoxic tumors, but recent studies suggest that HIF-1α can also induce antioxidant responses, mitigating ROS and promoting tumor survival. This feedback complicates the therapeutic use of HIF inhibitors and suggests a need for dual-targeting strategies.

#### 4.2.4. Role of TPA

According to studies, TPA can trigger the PKCα-MEK-ERK signaling pathway, induce the production of p16, and limit the development of human hepatoma cells [148]. TPA can additionally trigger EMT in human hepatoma cell lines. There appears to be a close relationship between ROS, TPA-mediated signaling, and EMT since studies have shown that ROS are essential for maintaining PKC-ERK signaling during TPA-induced EMT-like cell migration and dispersion in hepatoma cells. It was found that PKC is required for the ROS generation induced by TPA and that ROS scavengers, such as catalase and SOD, significantly reduce this effect. Mannitol, often used as a hydroxyl radical scavenger, also reduced TPA-induced cell motility, although its ROS-scavenging activity is nonspecific and primarily limited to •OH. TPA has also been demonstrated to change the activity of E-cadherin and integrins in a way that is dependent on PKC and ROS. These outcomes demonstrate that ROS are crucial for the sustained induction of PKC caused by TPA, which inhibits the production of E-cadherin and causes the EMT phenotype to emerge. This may be compared to preserving the biological characteristics of cancer stem cell reservoirs and specialization [149]. TPA-induced EMT via ROS and PKC/ERK signaling is well demonstrated, yet the dependence of this process on specific ROS types remains unclear. The variability in response to ROS scavengers points to complex intracellular redox compartmentalization that needs further clarification for translational relevance.

#### 4.2.5. Role of MMPs

MMPs belong to a family of enzymes responsible for the degradation of the extracellular matrix, and they are also known for playing a crucial role in facilitating tumor invasion. Tumor metastasis involves several mechanisms, including cell invasion, basement membrane deterioration, and stromal extracellular matrix degradation, which ultimately lead to the invasion and spread of tumor cells. It has been shown that increased MMP expression is predictive of tumor aggressiveness, metastasis, and poor patient survival in various human cancers, indicating that MMPs are critical for multiple facets of tumor aggressiveness [150].

MMPs play a significant role in initiating EMT. TGF-mediated activation of EMT was observed to promote MMP-28 expression in A549 lung adenocarcinoma cells [151]. The ability of MMP-3 to cause EMT and malignant transformation in cultured cells, as well as genomically fragile mammary carcinomas in transgenic mice, has been demonstrated. Loss of intact E-cadherin, enhanced mobility and metastatic dispersion, negative modulation of epithelial markers, and positive modulation of mesenchymal markers are some of the features of MMP-3-induced EMT. Additionally, the GTPase Rac plays a part in this process, indicating that Rac activation is a mediator of MMP-3-induced EMT [152]. Rac signaling is regarded as a key player in integrin signaling and is recognized to be necessary for cytoskeletal reorganization [151]. The integrin–Rac pathway can generate ROS, which cause tumor cells to move and invade, as demonstrated by a number of studies [153,154]. The production of ROS and enhanced Rac1b expression seem to be the processes that mediate MMP-3-induced EMT. It is interesting to note that the ROS-quenching compound acetyl cysteine (NAC) successfully suppressed MMP-3-induced EMT. These findings strongly imply that MMP-3 therapy enhances Rac1b expression, which raises intracellular ROS and induces EMT. Therefore, it may be possible to reduce tumor aggressiveness by reversing or eradicating EMT-type cells or cancer stem cells using MMP-3 inhibitors or ROS inhibitors [152]. While MMPs are established downstream effectors of ROS in metastasis and EMT, evidence also suggests that ROS can independently modulate EMT without MMP induction. The degree to which MMP-driven ECM remodeling versus ROS-driven transcriptional reprogramming predominates may vary with tumor type and stage.

#### 4.2.6. Further Factors Leading to EMT

The generation of ROS, which has been said to be closely related to EMT processes, is influenced by a variety of circumstances. Hepatocyte growth factor (HGF) has been demonstrated, for instance, to promote EMT and migration across a variety of cancer cells. This may involve the intricate process of ROS and enhance cancer cells’ capacity to spread across the entire body [155,156,157]. Furthermore, studies have shown that EGF can boost the migration of pancreatic cancer cells by activating and generating MMP-2 in a manner that is dependent on Rac1/ROS [158]. In rat hepatoma cells, blocking the EGF pathway increases TGF-induced apoptosis via inducing oxidative stress and changing the expression pattern of NOX isoforms [159]. ROS regulate the synthesis of VEGF and HIF-1 in human ovarian cancer cells by activating Akt and ribosomal S6 kinase1 p70 (p70S6K1). Furthermore, it has been shown that EGF can cause EMT in human cancer cell lines, indicating that more research is needed to determine whether ROS are necessary for EGF-induced EMT [44].

Studies have also revealed that miRNAs control crucial genes, which play a critical role in EMT, and ROS critically influence miRNAs [160,161]. Since their expression may serve as a regulator for maintaining the epithelial phenotype, it has been shown, for example, that the miR-200 family and miR-205 are significant regulators of EMT. The ZEB1/deltaEF1 and ZEB2/SIP1 transcriptional suppressors of E-cadherin and the miR-200 family are components of a signaling network, and the latter has been found to regulate PDGF-D-linked EMT in prostate cancer cells [162].

The contribution of additional ROS-regulated factors like EGF, HGF, and miRNAs in EMT underscores the complexity of redox signaling. However, the synergistic versus independent effects of these mediators remain poorly resolved, highlighting the need for integrated network-level analysis in EMT regulation.

### 4.3. ROS and Angiogenesis

Angiogenesis, a process that occurs in the early stages of carcinogenesis and produces new blood vessels from already existing vasculatures, enhances the growth and survival of tumors. Cancer proliferation triggers ROS-associated angiogenesis, which, in turn, accelerates metabolism and results in high levels of ROS [163,164]. These increased ROS levels cause the tumor microenvironment to experience oxidative stress, which starts the release of angiogenic modulators [165] via ROS-dependent cellular signaling, cytokines, growth factors, and transcription factors that are stimulated by both endogenous and exogenous ROS, thereby promoting tumor migration and proliferation [165,166]. Through hypoxia-independent or hypoxia-dependent mechanisms, it has been shown that the signaling cascade controlled by ROS can maintain VEGF secretion and activate the PI3K/Akt/mTOR pathway [167]. Moreover, it has been noted that the Ras signaling pathway increases VEGF release [168]. It was discovered that mutant p53 influences the angiogenic response in tumor growth by activating HIF-1 and VEGF-A in HCT116 human colon cancer cells via ROS [169]. To comprehend the signaling cascade that modifies the course of cancer, the process of ROS-mediated angiogenesis has been widely researched.

In another study, the epidermal growth factor (EGF) was found to increase hydrogen peroxide generation, which, in turn, triggers p70S6K1 through PI3K/Akt signaling, resulting in the downstream activation of VEGF and HIF-1 [170]. Similarly, some other studies suggested that, in ovarian cancer cells, EGF boosts the generation of hydrogen peroxide, which, in turn, triggers Akt/p70S6K1 signaling and enhances the expression of VEGF. The team also found that rapamycin and catalase overexpression prevent angiogenesis [44]. Moreover, it was shown that hydrogen peroxide can activate the PI3K/Akt/mTOR signaling cascade and Ras and inactivate PTEN by reversibly oxidizing phosphatase at the cysteine thiol group [171].

Another study using ovarian cancer cells demonstrated that NOX4 knockdown decreases HIF-1 and VEGF, which, subsequently, in response, regulates tumor angiogenesis [172,173] in melanoma cells, showing that ROS operate through a similar mechanism, while Akt stimulates the production of NOX4 [173,174]. It has also been demonstrated that ERK/PI3K/Akt/Src (Proto-oncogene tyrosine-protein kinase) signaling controls the growth and dissemination of cancer. This system stimulates endothelial cells and causes angiogenesis by NOX2-derived ROS [175,176,177]. In CaCO-2 colon cancer cells, Ras/ERK-linked phosphorylation and stimulation of Sp1 by NOX1 have also been discovered to influence the overregulation of VEGF levels and angiogenesis [178,179]. In human umbilical vein endothelial cells (HUVECs), angiopoietin-1 (Ang1) activates p44/42, MAPK, and Tie-2 endothelial-specific tyrosine kinase receptors, resulting in transient ROS, which leads to vascular remodeling [42].

Additionally, it has been demonstrated that copper stimulates the EGFR/ERK/c-Fos pathway in hepatocellular carcinoma and breast cancer cells, augmenting ROS-mediated VEGF, HiF-1, and G-protein estrogen receptor (GPER) expression [180,181]. Similar to this, the cadmium-stimulated ERK/Akt pathway enhances the production of ROS and HiF-1, a downstream pro-angiogenic protein, in bronchial epithelial cells (BEAS-2B) [68]. Furthermore, it has been demonstrated that ROS control several transcription factors and extracellular remodeling proteins, such as VEGF, p53, HIF-1, and MMPs [182,183,184,185,186,187]. Moreover, several investigations using a plethora of cancer cells have documented that ROS increase the production of HiF-1 and angiogenesis by activating the PI3K/Akt signaling cascade [188,189,190].

#### 4.3.1. The VEGF-VEGFR2 System

Cys residues may undergo irreversible hyper-oxidation under elevated ROS conditions, leading to the formation of sulfinic and sulfonic acid derivatives, especially when the Trx and Grx systems are impaired. Studies using “redox-dead” Cys17Ser PKARIα knock-in mutant mice demonstrated that VEGF, tumor, and ischemia-induced angiogenesis are mediated by PKARIα oxidation and dimerization-mediated activation [191]. However, in quiescent epithelial cells (ECs), the cytosolic receptor tyrosine kinase domain of VEGFR2, which has two oxidation-sensitive Cys residues, is maintained in a reduced form by an antioxidant enzyme known as PRX-2. On the other hand, inactive VEGFR2, which cannot function on VEGF, is caused by Prx2 deficiency in dormant ECs, which increases Cys oxidation of VEGFR2 and results in the formation of disulfide bonds. Thus, to promote a ROS-dependent VEGF-VEGFR2-stimulated angiogenic transition, quiescent ECs must be maintained in a decreased condition [192].

It has been shown that the thiol oxidoreductase protein disulfide isomerase (PDIA1) maintains the redox-sensitive Cys domains of the mitochondrial fission protein Drp1 in a reduced state in dormant ECs. Diabetes-related PDIA1 depletion promotes Drp1 Cys-OH synthesis at the mitochondria-associated membrane (MAM), which leads to mitochondrial fragmentation and elevated production of mitoROS. These events impede angiogenesis and expedite the senescence of ECs. This suggests that the stability of quiescent ECs depends on oxidoreductase PDI’s redox control of the ER-mitochondria crosstalk at MAM. This may be necessary to fuel effective angiogenic reactions brought on by VEGF, an angiogenic growth factor that generates ROS [193].

ROS regulation of VEGFR2 is critical for angiogenesis, yet redox control of VEGFR2 activation shows contrasting behavior in quiescent versus activated endothelial cells. This suggests that therapeutic ROS modulation must consider endothelial heterogeneity to avoid impaired vascular homeostasis.

#### 4.3.2. The Role of HIF in Angiogenesis

Angiogenesis is crucial for the growth and progression of solid tumors because tumor cells do not receive enough oxygen and nutrients. Finally, vasculogenesis and/or neo-angiogenesis need to be triggered for a tumor to grow beyond a certain size. This capability of tumor cells is due to the “angiogenic switch,” a phenomenon that occurs when the balance of pro-angiogenic factors exceeds that of anti-angiogenic factors. Pro-angiogenic factors that can be expressed as a result of HIF include vascular endothelial growth factor (VEGF), VEGF receptors FLT-1 and FLK-1, platelet-derived growth factor B (PDGF-B), plasminogen activator inhibitor-1 (PAI-1), the TIE-2 receptor, matrix metalloproteinases (MMP-2 and MMP-9), and angiopoietins (ANG-1 and ANG-2) [194].

VEGF-A, a potent endothelial mitogen that is present in many human malignancies, is the most remarkable protein among all of these HIF-activated pro-angiogenic factors [195,196]. HIF-1 is crucial to the biology of endothelial cells (ECs) and angiogenesis. Loss of HIF-1 hinders EC angiogenesis, which involves proliferation, migration, and chemotaxis, including wound healing [197]. Some studies found that HIF-2 positively influences the production of VEGF in RCC cells lacking VHL [198]. Some other studies demonstrated that PAI-1 is increased by HIF-2 to limit active plasmin, which, in turn, enhances angiogenesis in HepG2 cells [199]. These results imply that HIF-1 and HIF-2 are both involved in tumor angiogenesis. Although HIF-1α and HIF-2α promote angiogenesis under oxidative stress, their contributions may diverge depending on tumor type and microenvironmental conditions. Moreover, the precise role of ROS in modulating their transcriptional activity is still under debate, especially in VHL-deficient versus wild-type tumors.

#### 4.3.3. The Role of p66Shc in Angiogenesis

Adaptor protein p66Shc is one of the main regulators of mitoROS in relation to the ETC. p66shc translocates to the mitochondria, where it oxidizes cytochrome c to produce H_2_O_2_ after being phosphorylated at the Serine (Ser) 36 residue in the cytoplasm. By interacting via a non-phosphorylated form of p66shc, VEGF activates Rac1 quickly. This causes NOX2-dependent ROS to be produced, which, in turn, causes VEGFR2 to be phosphorylated at caveolae/lipid rafts and the following angiogenic responses in ECs [200]. Moreover, it has been demonstrated that p66shc controls the expression of the cytosolic NOX organizer p47phox, which in turn controls ROS production [201,202]. It has also been reported that VEGF increases the amount of p66Shc that is phosphorylated at Ser36 via protein kinase C (PKC) and ERK/Jun N-terminal kinase (JNK), both of which are activated by NOX-derived H_2_O_2_. It consequently promotes prolonged ROS-dependent VEGFR2 signaling and angiogenic responses by increasing the generation of mitoROS [203]. Overall, angiogenesis under redox stress emerges as a tightly controlled, ROS-amplified cascade modulated by oncogenic cues and hypoxia. Yet, contradictory reports on VEGF–ROS interactions across different tumors suggest that ROS-driven angiogenesis is not uniform and must be interpreted in the context of tumor stage, vascular density, and microenvironmental cues.

### 4.4. Cutting-Edge Tools for Monitoring Redox Signaling Heterogeneity

ROS exhibit multifaceted biological functions spanning cellular signaling pathways, proliferation regulation, and apoptotic mechanisms. Comprehensive characterization of these dynamic molecules through precise quantification methods is essential for elucidating their diverse physiological contributions [204].

Despite the inherent difficulties in designing reliable tools for the detection and quantification of ROS, significant progress has been made through the development of diverse analytical approaches. Techniques employing absorbance measurements, fluorescence-based assays, chemiluminescent detection [205], and genetically encoded biosensors [204] have been extensively explored and optimized for monitoring ROS under various experimental conditions.

Genetically encoded biosensors offer powerful tools to visualize redox fluctuations in living cells with high resolution. Contemporary technological developments are synthesized from approximately six principal biosensing platforms that demonstrate significant relevance for oxidative species measurement in oncological research models [206].

Redox-sensitive fluorescent proteins are engineered from green fluorescent protein or yellow fluorescent protein variants through the strategic insertion of cysteine residues, enabling disulfide bond formation upon oxidation. This structural shift alters fluorescence properties, allowing ratiometric or intensity-based detection of intracellular redox states. Variants like redox-sensitive yellow fluorescent protein and redox-sensitive green fluorescent proteins 1 and 2 have been optimized for dynamic range and compartmental targeting (i.e., mitochondria, endoplasmic reticulum, cytosol), and display real-time responsiveness to glutathione redox potential. Notably, redox-sensitive green fluorescent protein 2’s serine 65-to-threonine mutation enhances fluorescence excitation efficiency, making it suitable for imaging ROS in different oxidative environments. Further engineering of these probes, such as in the redox-sensitive green fluorescent protein 1-iX family, enhances their kinetics and thermodynamic range to better suit cancer-relevant compartments, like the endoplasmic reticulum and mitochondrial intermembrane space [206,207].

Fusion constructs of redox-sensitive fluorescent proteins with redox-active proteins enhance specificity and responsiveness. For example, glutaredoxin 1–redox-sensitive green fluorescent protein 2 and redox-sensitive yellow fluorescent protein–glutaredoxin 1 leverage glutaredoxin’s catalytic activity for rapid and glutathione-specific redox detection. This approach facilitates sub-second resolution of ROS dynamics. Other notable constructs include redox-sensitive green fluorescent protein 2–oxidant receptor peroxidase 1, for hydrogen peroxide detection via thiol peroxidase coupling, and mycoredoxin 1–redox-sensitive green fluorescent protein 2 or bacilliredoxin–redox-sensitive green fluorescent protein 2, which are adapted for organisms with alternative thiol systems (for example, mycothiol or bacillithiol). These tools have been applied in diverse cellular models and are particularly relevant in dissecting oxidative signaling in cancer cells with altered thiol homeostasis [206,208].

Light–oxygen–voltage domains, derived from plant and microbial signaling proteins, exhibit blue-light-responsive fluorescence via a flavin mononucleotide chromophore. Mutant versions—like improved light–oxygen–voltage domain (cysteine 426 to alanine)—are repurposed as redox sensors due to their reversible electron acceptance properties. The pH and redox optical sensor probe, a dual-emission chimera of improved light–oxygen–voltage domain and monomeric blue-emitting red fluorescent protein, enables simultaneous pH and redox state tracking. Fusion with glutaredoxin 1 (glutaredoxin 1–pH and redox optical sensor) enhances specificity toward glutathione-based changes. These compact, photostable, and oxygen-independent probes are ideal for redox analysis under hypoxic or fluctuating pH conditions typical in tumor microenvironments [206,209].

Peroxiredoxins are abundant cellular antioxidants that undergo oxidation-induced dimerization via intersubunit disulfide bonds. Förster resonance energy transfer-based probes integrating peroxiredoxin 2 between fluorescent protein pairs (for example, peroxiredoxin–Clover–monomeric Ruby fluorescent protein 2) capture hydrogen peroxide-mediated dimerization events. These biosensors are highly selective for hydrogen peroxide and show negligible pH interference—suitable for cancer cells where transient ROS bursts regulate signal transduction. Bacterial and fungal peroxiredoxin-based sensors have also been developed using circularly permuted fluorescent proteins to extend ROS quantification to diverse microbial models or subcellular niches [206,210,211].

HyPer sensors, based on circular permutation of yellow fluorescent protein fused to the OxyR regulatory domain, specifically report hydrogen peroxide levels. The oxidation-induced disulfide formation triggers a conformational change that modulates circularly permuted yellow fluorescent protein fluorescence. Successive generations (HyPer-2, HyPer-3, HyPer-7) have improved response speed, brightness, and pH stability. HyPer-7, for instance, offers ultrafast and ultrasensitive detection, with a significantly enhanced extinction coefficient and pH-independence, making it highly suitable for live imaging in tumor microenvironments. Additional constructs, such as the triresistance peroxidase sensor and the Neon fluorescent protein oxidation indicator for irradiated environments, extend HyPer utility to oxidizing compartments, like the endoplasmic reticulum, or maintain signal stability across wide pH ranges—key for tumor biology applications. Thioredoxin–red fluorescent protein 1 to 3 series, exploiting circularly permuted red fluorescent protein and thioredoxin fusions, allow for selective monitoring of thioredoxin pathway activity in redox-driven malignancies [206,212].

The yeast transcription factor yeast activator protein 1 undergoes redox-sensitive conformational changes mediated by disulfide bond formation in response to hydrogen peroxide. This mechanism is exploited in Förster resonance energy transfer-based probes (for example, oxidative Förster resonance energy transfer, peroxide Förster resonance energy transfer, and redox fluorescence resonance energy transfer), where oxidative rearrangement alters the spatial configuration of fluorophore pairs, yielding a quantifiable signal. These probes have been used to study compartment-specific ROS levels and can be adapted to model ROS-driven transcriptional responses in cancer cells. In parallel, synthetic transcriptional biosensors using yeast activator protein 1 response elements upstream of fluorescent reporters offer a means to monitor oxidative stress-induced gene expression [206,213,214].

Genetically encoded ROS biosensors represent a powerful and expanding toolkit for tracking redox dynamics with spatial and temporal precision. Their utility in cancer research is significant, offering insight into ROS-mediated signaling, oxidative damage, and antioxidant responses. As new biosensor designs continue to emerge, improving on specificity, kinetic response, and stability, they pave the way for dissecting redox mechanisms in tumor biology and therapy resistance with unprecedented detail [215,216].

An optimal imaging methodology for ROS should enable precise detection of concentration fluctuations with both high spatial and temporal resolution. It must also discriminate among distinct redox pairs and be sensitive to changes occurring within physiological concentration ranges. For successful in vivo implementation, additional factors such as sensor bioavailability, effective tissue penetration, and a robust signal-to-noise ratio are essential considerations [217].

Recent advancements, such as autofluorescence multispectral imaging (AFMI), address the limited real-time, non-invasive detection of ROS dynamics in cancer cells. By leveraging endogenous fluorophores sensitive to redox changes, AFMI quantifies ROS without perturbing cellular function. This technology enables single-cell resolution and spatial mapping, revealing intra- and intercellular ROS heterogeneity previously undetectable with conventional methods. As a result, AFMI reshapes our understanding of redox signaling variability within tumors, offering new insights into metabolic adaptation, therapy resistance, and tumor progression, and holds strong translational potential for real-time monitoring in clinical oncology [218].

Surface-Enhanced Raman Spectroscopy (SERS) has emerged as a powerful technique for detecting intracellular ROS in cancer biology due to its high sensitivity, narrow spectral peaks, and low interference from water. By enhancing the Raman signal of target molecules through localized surface plasmon resonance on metallic nanoparticles, SERS enables the detection of ROS species, such as peroxynitrite (ONOO^−^) and hypochlorite (ClO^−^), at low concentrations in living cells. In this approach, AuNPs functionalized with specific ligands (4-MPBA and 2-MP) selectively react with ROS to produce distinct Raman spectral changes. The integration of SERS with deep learning models (ENN and 1D-CNN) allows accurate spectral feature extraction and quantification of ROS in situ. This combination overcomes traditional challenges such as overlapping signals and manual interpretation, enabling real-time monitoring of oxidative dynamics in heterogeneous tumor environments. Consequently, SERS-based platforms offer novel insights into redox signaling pathways in cancer, aiding early diagnosis and understanding of treatment resistance mechanisms [219,220].

Europium-doped gadolinium orthovanadate nanoparticles represent an advanced tool for real-time, spatially resolved ROS imaging in cancer biology. Capable of detecting H_2_O_2_ dynamics at nanometer resolution and second-scale timeframes, they allow quantitative monitoring in the micromolar to millimolar range without signal saturation. These nanosensors reveal heterogeneity in ROS signaling, such as stimulus-specific H_2_O_2_ gradients in vascular smooth muscle cells, underscoring the spatial complexity of redox signaling. These nanoparticles respond to a range of strong oxidants yet can selectively detect H_2_O_2_ under controlled conditions. Their tunable excitation properties enable flexible quantitative analysis, and their stability across varying ROS concentrations allows prolonged monitoring. Importantly, they maintain functionality in complex intracellular environments, making them powerful tools for studying redox dynamics in cancer contexts. Their low cytotoxicity and in vivo compatibility further support their potential for studying oxidative processes in tumor microenvironments, providing critical insights into inflammation, signaling pathways, and therapeutic responses [221].

A newly optimized chemiluminescence-based method allows real-time quantification of low concentrations of ROS in nonphagocytic cancer cells, such as HT-29 colon carcinoma. This technique employs luminol dissolved in sodium hydroxide and is enhanced by 4-iodophenylboronic acid to improve both the rate and intensity of the hydrogen peroxide-induced chemiluminescent signal. Using a chemiluminescent imaging system, it enables parallel measurement of intracellular and extracellular ROS with micromolar sensitivity. Importantly, the system reveals dynamic changes in ROS levels and their correlation with tyrosine phosphorylation of protein tyrosine kinases, like focal adhesion kinase, proline-rich tyrosine kinase 2, and Src. These findings indicate that low concentrations of hydrogen peroxide regulate signaling heterogeneity through redox-sensitive phosphorylation events. By enabling spatially and temporally resolved analysis, this method significantly improves our understanding of redox-dependent signaling pathways in cancer biology and offers a cost-effective, high-throughput alternative to fluorescence-based assays [205].

## 5. Tumor Suppressive Role of ROS

As was previously established, cancer cells have higher levels of ROS than healthy ones. Yet, these ROS levels are reduced by cancer cells’ elevated antioxidant enzyme activity. This shared impact leads to the assumption that therapeutic strategies that either increase ROS production or decrease antioxidant defense may overstress cancer cells, activating a number of cell death pathways and arresting the progression of the disease [222]. In reality, a large variety of medicines against cancer effectively destroy cancer cells and render certain cancer cells susceptible to chemotherapy medications. In the sections that follow, the significant roles that ROS play in tumor suppression are highlighted, as listed in Table 2.

### 5.1. Apoptosis

In multicellular organisms, apoptosis, sometimes referred to as organized cell death or type I programmed cell death, is the most prevalent and essential way of destroying cells. It is a closely controlled and coordinated system that plays an important role in maintaining tissue homeostasis, eliminating damaged or undesirable cells, and contributing to numerous physiological processes, such as embryonic development and immunological response. At the core of apoptosis lies a distinct family of proteases known as caspases. Caspases are cysteine-dependent, aspartate-directed proteases that start and complete the apoptotic process. When caspases are activated, a series of proteolytic activities begin, which finally result in the breakdown of organelles and the controlled disintegration of the cell. The extrinsic (depending on death receptors) and the intrinsic (mitochondrial dependent) routes are the two main, well-studied apoptotic processes. Death-inducing ligands, like Fas ligand (FasL), TNF, and TNF-related apoptosis-inducing ligand (TRAIL), bind to their corresponding receptors, which include the Fas receptor (FasR), the TNF receptor (TNFR), and the death receptors (DR4 and DR5), to mediate the extrinsic pathway of apoptosis. The ligand–receptor interactions result in the formation of the death-inducing signaling complex (DISC), which also recruits adaptor proteins (FADD and TRADD) and pro-caspase (mostly procaspase-8), triggering the caspase cascade and resulting in apoptosis. Physically linked to mitochondria, the intrinsic apoptosis pathway—which contains conserved signaling proteins—is also vulnerable to mitochondrial oxidative stress in vertebrates. Members of the Bcl family linked to the mitochondrial membrane, such as Bax and Bcl-2 gene, which function as pro- or anti-apoptotic regulatory proteins, affect the mechanism [306,307,308].

**Figure 4:**
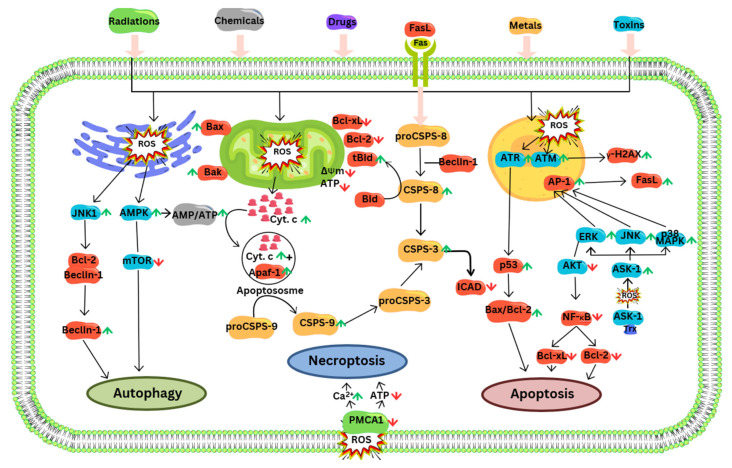
Thioredoxin (Trx) oxidation and apoptosis signal-regulating kinase 1 (ASK1) activation are caused by an increase in ROS levels in the cytoplasm. ASK1 phosphorylates mitogen-activated protein kinases (ERKs, JNKs, p38 MAPKs), which, in turn, regulate the production of death ligands (FasL), pro-apoptotic proteins (Bak, Bax), anti-apoptotic proteins (Bcl-2, Bcl-xL), and pro-apoptotic proteins (Bcl-2, Bcl-xL). In this way, ERKs encourage cell death by reducing the activity of AKT kinase, which regulates the creation of Bcl-2 and Bcl-xL proteins via NF-κB. The oxidative damage to the mitochondria causes the release of cytochrome c (Cyt. c) into the cytosol, reduction in ATP levels, and dissipation of mitochondrial membrane potential. Caspase-dependent apoptosis is brought on by Cyt. c’s interaction with apoptotic protease activating factor 1 (Apaf-1). Ataxia telangiectasia mutated (ATM) and ataxia telangiectasia and Rad 3-related (ATR) kinases are activated because of an increase in ROS levels in the nucleus, which causes p53-mediated apoptosis and is responsible for the rise in the DNA double-strand breaks marker (H2AX) level. Beclin-1, a pro-autophagic protein, is upregulated by JNK1 in response to the ROS-induced endoplasmic reticulum stress, whereas mTOR kinase, an inhibitor of autophagy, is downregulated by AMPK. Both procedures result in autophagy of the cell. The downregulation of plasma membrane calcium ATPase 1 (PMCA1), a decrease in ATP levels, and an increase in calcium ion levels are all consequences of oxidative damage to the plasma membrane. As a result, cell necrosis happens next. JNK1, c-Jun N-terminal kinase 1; Bcl-2, B-cell lymphoma 2; mTOR, mammalian target of rapamycin; AMPK, AMP-activated protein kinase; ROS, reactive oxygen species; AMP/ATP, AMP/adenosine triphosphate ratio; Bax, Bcl-2-associated X protein; Cyt. c, cytochrome c; Apaf-1, apoptotic protease-activating factor 1; proCSPS-9, pro-caspase-9; CSPS-9, caspase-9; Bcl-xL, B-cell lymphoma-extra-large; ATP, adenosine triphosphate; ΔΨm, mitochondrial membrane potential; CSPS-8, caspase-8; CSPS-3, caspase-3; proCSPS-8, procaspase-8; FasL, Fas ligand; Fas, Fas receptor; PMCA1, plasma membrane calcium-transporting ATPase 1; ICAD, inhibitor of caspase-activated DNase; p53, tumor protein p53; NF-κB, nuclear factor kappa-light-chain-enhancer of activated B cells; ASK-1, apoptosis signal-regulating kinase 1; Trx, thioredoxin; AKT, protein kinase B; ERK, extracellular signal-regulated kinase; p38MAPK, p38 mitogen-activated protein kinase; AP-1, activator protein 1; γ-H2AX, phosphorylated histone H2AX; ATM, ataxia-telangiectasia mutated; ATR, ataxia telangiectasia and Rad3-related.

Various compounds have been reported to induce ROS-mediated apoptosis. Several curcumin analogues have been assessed for their effectiveness against HepG2 cells for cytotoxicity. In 3,3′-OH curcumin, the hydroxyl group at 3,3′-position is crucial in boosting anti-proliferation action via the production of ROS, like hydroxyl radicals and H_2_O_2_ in HepG2 cells. The production of ROS can cause lipid peroxidation, leading to the collapse of the membrane potential of the mitochondria and ultimately to apoptosis. Additionally, it may affect the intracellular redox equilibrium [309] (Figure 4).

Similarly, a natural polyphenolic alkanone (6)-gingerol (6G) also possesses anti-inflammatory and anticancer effects. It was found that 6G treatment led to the generation of ROS in U937 and K562 cell lines through the inhibition of the mitochondrial respiratory complex I (MRC I), which, in turn, raised the expression of the oxidative stress response-related microRNA miR-27b and DNA damage. Increased miR-27b expression reduced PPARγ, which, in turn, reduced the expression of inflammatory cytokines linked to the oncogenic NF-κB pathway. Meanwhile, increased DNA damage caused G2/M cell cycle arrest [310].

The homovanillic acid derivative known as capsaicin suppressed the growth of leukemic cells, but not normal bone marrow mononuclear cells, via the induction of G_0_–G_1_ phase cell cycle arrest and apoptosis. Apoptosis caused by capsaicin was followed by a spike in the intracellular generation of ROS in the presence of wild-type p53 capsaicin-sensitive leukemic cells. The phosphorylated p53 at the Ser-15 residue was observed. By preventing the phosphorylation of p53’s Ser-15 residue, the antioxidants N-acetyl-l-cystein and catalase, but not superoxide dismutase, fully prevented capsaicin’s ability to cause apoptosis [311].

*Rosmarinus officinalis* L.’s primary antioxidant, carnosic acid (CA), has been shown to have anticancer properties. It was found that in HCT116 cells, CA administration induced apoptosis, followed by elevated caspase-9, caspase-3, p53, and Bax. It also suppressed Mdm2, Bcl-2, and Bcl-xl and led to breakage of PARP. Moreover, colon cancer cells produced ROS following CA therapy. The ROS scavenger N-acetyl cysteine reversed the inhibitory action of CA on the JAK2-STAT3/Src-STAT3 pathway and shielded cells from CA-induced mortality by inhibiting the activation of p53 and the destruction of caspase-3 and PARP in HCT116 cells. However, the therapeutic inhibitor of GSH synthesis, L-buthionine-sulfoximine, boosted the ROS production that CA caused, potentiating the apoptotic impact of CA. Therefore, it was concluded that CA caused death in HCT116 cells by producing ROS, upregulating p53, activating caspases, and inhibiting the STAT3 signaling pathway [312].

Cepharanthine is a natural compound derived from medicinal plants with strong anti-cancer properties. Lung cancer cells undergo apoptosis induction following cepharanthine’s production of ROS and the loss of the potential of the mitochondrial membrane. It is interesting that ROS are involved because these effects were greatly reduced when cells were pre-treated with N-acetylcysteine (NAC), a specific ROS inhibitor [313].

#### 5.1.1. PI3k Pathway

The ability of some natural compounds to induce cell death in cancer cells, such as cedrol and isoorientin, has been investigated. Cedrol was found to initiate cell death in A549 human lung carcinoma cells through a multifaceted process. Firstly, cedrol treatment activated the PI3K/Akt pathway, but, interestingly, it led to the suppression of Akt phosphorylation, suggesting inhibition of this survival pathway. Cedrol simultaneously caused mitochondrial malfunction, leading to a reduction in mitochondrial transmembrane potential and oxidative stress by producing ROS. The intrinsic apoptotic pathway was then set into action when pro-apoptotic molecules were released from the mitochondria, which resulted in the activation of caspase and cell death. Cedrol also promoted autophagy, which was initially investigated to be a protective mechanism, but, eventually, the interaction between autophagy induction, mitochondrial malfunction, and ROS generation eventually led to apoptotic cell death in A549 cells. On the other hand, isoorientin triggered apoptosis in cancer cells by causing the production of ROS, which caused oxidative stress. The increased ROS levels disrupted ΔΨm and caused the release of cytochrome c, activating caspase-9 and subsequently caspase-3, ultimately leading to DNA fragmentation and cell death. To further promote mitochondrial malfunction and apoptosis, isoorientin also decreased the anti-apoptotic protein Bcl-2 and increased the pro-apoptotic protein Bax. Moreover, in 5637 bladder cancer cells, fucoidan induced apoptosis by downregulating telomerase activity and inactivating the PI3K/Akt pathway. The increased ROS levels resulted in the downregulation of telomerase activity, leading to telomere shortening, while the PI3K/Akt pathway was deactivated, thus shifting the balance towards cell death. The combined effects of ROS generation, telomerase activity downregulation, and PI3K/Akt pathway inactivation ultimately led to apoptotic cell death in 5637 bladder cancer cells. These findings provide insights into the diverse molecular mechanisms through which cedrol, isoorientin, and fucoidan induce cell death in cancer cells and highlight their potential as therapeutic agents against cancer [239,256,273].

#### 5.1.2. MAPK Pathway

Three natural substances, including bruceine D, 7-O-geranylquercetin, and eupatilin, were investigated by many researchers in diverse cancer cell types. The first substance, bruceine D, caused oxidative stress and the subsequent activation of the MAPK signaling pathway, which led to cell death in lung cancer cells by causing the production of ROS. Increased ROS levels caused MAPK proteins (ERK, JNK, and p38) to become phosphorylated, mediating cellular responses to stress and eventually leading to apoptotic cell death. Caspase-3 activation and PARP cleavage demonstrated the presence of apoptotic signaling pathways. Similar to this, 7-O-Geranylquercetin caused gastric cancer cells to die by raising intracellular ROS levels, activating the MAPK pathway, and causing oxidative stress. As a result, pro-apoptotic factors like cytochrome c were released, caspase-9 and caspase-3 were activated, and apoptotic cell death was subsequently started. These events led to mitochondrial dysfunction, which was indicated by a decreased mitochondrial membrane potential (m). The modulation of the Bcl-2 and Bax proteins further promoted mitochondrial collapse and death. In the case of eupatilin, ROS production and subsequent activation of the MAPK signaling pathway led to the induction of cell death in renal cancer cells. Increased levels of cleaved caspase-3 and PARP indicated that activation of MAPK proteins caused apoptotic cell death. Eupatilin treatment additionally impacted the PI3K/AKT signaling pathway, reducing AKT phosphorylation, which probably turned the balance in favor of cell death as opposed to cell survival and proliferation [230,237,268].

### 5.2. Autophagy

A process that involves several stages, termed autophagy, recycles damaged organelles, intracellular aggregates, and long-lasting proteins to maintain and restore cellular homeostasis. This procedure necessitates the involvement of roughly 40 proteins and results in the production of the phagophore, a double-membrane structure that engulfs organelles as well as a portion of the cytoplasm to create an autophagosome. The lysosome and this vesicle eventually merge into an autophagolysosome, which causes the content to break down and be recycled [314]. Autophagy has been shown to be dysregulated in a variety of illnesses, particularly malignancies and neurological diseases, and it has been reported to play a role in biological processes, including development, homeostasis, and cellular immune response [315]. Although autophagy is now well acknowledged to play a defensive function in neurodegenerative illnesses, its impact on cancer is far more nuanced and paradoxical. The role of autophagy in the growth of a tumor can be favorable or negative, depending on the stage of the tumor and the tissue or cells targeted. For instance, autophagy can stop healthy cells from becoming malignant, but it can also make cells resistant to chemotherapeutic drugs. More significantly, this beneficial or detrimental effect appears to be closely tied to the amount of autophagy caused by various intracellular or extracellular stressors, like ROS build-up [316,317,318].

The key regulator of the initiation of phagophore creation during the activation of autophagy remains the mammalian target of rapamycin complex 1 (mTORC1) kinase, which can integrate numerous external stimuli. After mTORC1 is suppressed, PI3K and Unc-51 like autophagy activating kinase 1 (ULK1)/ATG1 are triggered to begin the development of a phagophore. Then, two post-translational changes that are comparable to ubiquitinoylation or SUMOylation are activated, lengthening the double-membrane structure and causing the covalent attachment of ATG12 onto ATG5 and ATG8 family members onto phospholipids. Finally, it is acknowledged as a direct indicator of intracellular autophagy because it is thought to be connected with autophagy levels [319].

Diosgenin, a steroidal saponin isolated from legumes and yams, which, on treatment, causes an increase in mitochondrial ROS, inhibiting mTORC1 and inducing autophagy. Repression of mTORC1 in response to oxidative stress can be alleviated with the ROS scavenger NAC. The mTOR signaling pathway regulates protein translation positively by phosphorylating p70S6K and 4E-BP1 and negatively by phosphorylating mTORC1 at serine 2448. Diosgenin-triggered ROS-dependent autophagy and cytotoxicity rely on the mTOR signaling pathway, which is critical [291].

Similarly, DOP (*Dendrobium officinale* polysaccharide) acts by disrupting mitochondrial function, reducing ATP/energy charge, altering mitochondrial ROS production, and causing MMP loss. AMPK signaling is activated in the presence of low energy, and activated AMPK inhibits mTORC1. DOP significantly reduces mTOR phosphorylation while increasing AMPK expression and phosphorylation, implying that DOP activates AMPK-mTOR autophagy signaling, leading to cell death [298].

SZC017, an oleanolic acid derivative, stimulates autophagy following the overexpression of autophagy-related proteins, such as Beclin-1 and LC3-II, when administered. As a result, the activation of autophagy promotes the production of ROS. The Akt and JAK2/STAT3 signaling pathways are both involved in apoptotic cell death. SZC017 inhibits Akt phosphorylation, resulting in downregulation of the Akt signaling pathway, which is essential for the survival and proliferation of cells. Furthermore, it inhibits JAK2 and STAT3 phosphorylation, inactivating the JAK2/STAT3 signaling pathway, which is linked to cell survival and proliferation. After serving as a protective mechanism, SZC017-induced autophagy also contributes to apoptotic cell death by promoting the accumulation of mitochondria that are damaged, leading to ROS generation [295].

#### ROS-Induced Autophagy in Cancer Cells

A pentacyclic triterpenoid known as ursolic acid (UA) has been shown to have promising anticancer properties. The development of acidic vesicular organelles, a rise in autophagolysosomes, and LC3-II accumulation were indications that UA triggered autophagy. Moreover, it was discovered that UA causes ER stress, an increase in intracellular calcium, and the generation of ROS. The rise in free cytosolic calcium induced by UA stimulated the CaMKK-AMPK-mTOR kinase signaling pathway, which eventually induced autophagy [320].

Salinomycin, a polyether ionophore antibiotic, was found to cause cell death in human cancer cells that display a range of drug resistance pathways. Instead, autophagy-related characteristics, such as LC3 processing and cytoplasmic vacuolization, were visible in MCF-7 and SW620 cells. Caspase-capable cell lines induced autophagy prior to the commencement of caspase activation and at lower salinomycin doses. Salinomycin also increased the creation of ROS, which, in turn, activated JNK and generated the transcription factor JUN. N-acetyl-cysteine, a free radical scavenger, was able to partially halt salinomycin-induced cell death, suggesting that ROS generation is a key component in salinomycin toxicity [321].

A study showed that KIOM-C inhibited NF-κB-mediated MMP-9 activation in highly malignant cancer cells, which inhibited the spread of those cells. Cells were arrested in the G1 phase after treatment with KIOM-C. This was accompanied by an increase in p21 and p27, a decrease in cyclin D1, and a rise in apoptotic and autophagic cells. Apoptosis and autophagy were both effectively induced in cancer cells by KIOM-C through the activation of c-Jun N-terminal kinase (JNK) signaling pathways [322].

The administration of ciclopirox olamine (CPX) has been reported to cause ROS-induced cytoprotective autophagy in human rhabdomyosarcoma (Rh30 and RD) cells. It was shown that CPX and ROS activate the JNK, ERK1/2, and p38MAPK; however, only JNK was shown to be specifically involved in the induction of autophagy [323].

Another strategy that causes early ROS-induced autophagy and late apoptosis, which results in cell death, was studied in breast cancer cell lines utilizing the polyphenol carnosol. The authors demonstrated a relationship between mitochondrial damage, elevated ROS levels, autophagy, and cell death [324]. In this model, the scientists showed the close relationship between autophagy and apoptosis. They predicted that antioxidants and autophagy inhibitors would prevent the death of cancer cells. Similar findings were obtained using Resveratrol or psoralidin, which raised ROS production and autophagy levels, resulting in the death of human colon cancer (HT-29, COLO 201) and lung cancer (A549) cell lines, respectively. Antioxidants or the addition of 3-MA prevented these effects [325,326].

### 5.3. Necroptosis

Necroptosis is a unique type of controlled cell death that is distinct from apoptosis and necrosis. It is referred to as “programmed necrosis” or “regulated necrosis” because, like apoptosis, it requires specific signaling pathways and is not caused by random cell destruction. Necroptosis occurs in response to specific situations, particularly when apoptosis is hindered or unavailable [327,328]. Necrostatin-1 (Nec-1) is a pioneering necroptosis inhibitor that has been distinctly defined, and it is known to inhibit RIPK1 action alone. Necroptosis is primarily mediated by mixed lineage kinase domain-like pseudo-kinase (MLKL), receptor-interacting protein [RIP] kinase 1 (RIPK1), and RIPK3 [329].

The necroptosis signaling molecules RIP1, RIP3, and MLKL have been shown to be elevated when emodin triggers necroptosis. JNK, a stress-responsive protein, was activated by emodin treatment, and its inhibition reduced RIP1 and MLKL phosphorylation, indicating that it plays a role in emodin-induced necroptosis. Emodin also inhibited glycolysis in renal cancer cells by lowering glucose-6-phosphate, pyruvate, and ATP levels. This glycolysis inhibition was attributed to ROS, which reduced the expression of GLUT1, a critical glucose transporter protein. Furthermore, emodin inhibited PI3K/AKT signaling, resulting in lower GLUT1 expression. The authors hypothesized that emodin-induced GLUT1 downregulation via the ROS-mediated PI3K/AKT pathway played a role in inhibiting glycolysis and ultimately leading to cell death [330].

One study found that the emergence of mitochondrial ROS following endocytosis of selenium nanoparticles (SeNPs), a possible anticancer treatment, suggests that SeNPs damage mitochondria. After SeNP treatment, the expression of TNF and interferon regulatory factor 1 (IRF1) genes, which are associated with regulated necrosis (necroptosis), increased significantly. However, no RIP3 expression or phosphorylation of MLKL was observed, implying that the cell death mechanism induced by SeNPs may not involve the conventional RIP3-MLKL necroptosis pathway. Furthermore, SeNPs did not induce apoptosis in PC-3 cells. The study suggests that RIP3/MLKL-independent necroptosis may occur downstream to RIP1 in RIP3 non-expressing PC-3 cells. The findings shed light on the novel mechanism of SeNP-mediated cell death as well as its potential as a cancer chemo-preventive agent [331].

Neoalbaconol (NA), a necroptosis-inducing component isolated from *Albatrellus confluens*, has been shown to contribute to cell death via TNF-α feed-back signaling. The NF-κB pathway was activated after NA treatment, which prevented RIPK1 from being ubiquitinated. This was accomplished by downregulating TNF-α receptor-associated factors (TRAFs), cellular inhibitors of apoptosis protein 1/2 (cIAP1/2), and E3 ubiquitin ligases. The non-canonical NF-κB pathway was activated, stimulating the generation of TNF-α and inducing cell death. Additionally, NA enhanced cell death by enabling ROS to be produced in a RIPK3-dependent manner [332].

Arctigenin, a mitochondrial complex I inhibitor, was reported to cause necroptosis through ROS-mediated mitochondrial damage and cell communication network factor 1 (CCN1) upregulation. Moreover, CCN1 knockdown decreased MLKL, Bcl-2, and Mcl-1 levels, increased Bax, and caspase-3 cleavage. Interestingly, the sequence of arctigenin-induced events was successfully reversed by pretreatment with the ROS scavenger N-acetylcysteine, indicating that ROS acted as upstream molecules in arctigenin-driven cytotoxicity. These findings suggest that arctigenin may be a potential therapeutic agent for cancer cells by inducing necroptosis [333].

#### Role of RIPK1 in Signaling Necroptosis

An innovative strategy to control cancer by necroptosis requires a greater knowledge of the processes of necroptosis due to the growing importance of necroptosis in cancer. The necroptotic cell death pathway is thought to be activated by a wide range of stimuli, including members of the tumor necrosis factor receptor (TNFR) superfamily, T cell receptors (TCRs), pattern recognition receptors (PRRs), and several chemotherapeutic medicines [334]. Necroptosis can be brought on by environmental conditions such as hypoxia, which may be prevented by increased anaerobic glycolysis and glucose absorption in cancer cells [335].

The TNFα/TNFR signaling pathway is thought of as a prototype among the numerous stimuli and has received the most attention [336]. Hence, the activities that take place in the TNF signaling pathway might be thought of as the beginning of necroptosis. The recruitment of several proteins, including RIPK1, cIAP1, TRADD, cIAP2, TRAF2, and TRAF5, occurs when TNF binds to TNFR1. RIPK1, a crucial regulator of cell destiny that is polyubiquitinated by cIAP1/2, is a component of complex I, a membrane-bound multimeric protein complex. This, in turn, activates the NF-κB pathway, which transactivates cytoprotective genes and encourages cell survival. In addition, the rapid internalization of ligand-bound TNFR affects the proteins in complex I and their post-translational alterations [336,337,338]. For instance, cylindromatosis tumor suppressor protein (CYLD) is a deubiquitinase that deubiquitinates RIPK1, which restricts the prolonged activation of NF-κB signaling and tends to activate cell death pathways [337,339]. Consequently, complex II, commonly known as the “ripoptosome,” which includes RIPK1, caspase-8, TRADD, and FADD (FAS-associated death domain protein), a cytoplasmic death-inducing signaling complex, is created, activating caspase-8. Complex II regulates the activation of the necroptotic and apoptotic pathways [329,340]. RIPK1 and RIPK3 in complex II become inactive when active caspase-8 cleaves them. This starts the pro-apoptotic caspase activation cascade, which eventually results in the execution of apoptosis [341]. It has also been suggested that caspase-8 increases cell survival by cleaving CYLD. Nevertheless, RIP kinase cleavage ceases when caspase-8 is inhibited by medication or genetic modification, and the cell death process is switched to necroptosis [337,342] (Figure 4).

The autophosphorylation of serine residue 161 (S161) at RIPK1’s N-terminal causes it to be phosphorylated and activated once the cell death mode is altered [328]. When activated, RIP1 interacts with RIPK3 through their respective RIP homotypic interaction motifs (RHIMs), and the necrosome complex—a critical molecular signaling platform for necroptosis—is created [336]. The activation of RIPK1 autophosphorylation by mitochondrial ROS was discovered to cause RIPK3 recruitment. ROS generation also necessitates the presence of RIPK3 in necrosomes, resulting in a positive feedback loop that efficiently induces necroptosis [336,343]. According to one report, CYLD activates necrosomes by deubiquitinating RIPK1 following necrosome construction. Numerous RIPK3 inhibitors, including the RIPK1 inhibitor nec-1 and the MLKL inhibitor necrosulfonamide (NSA), among others, can stop necrosome formation and/or activation [344,345]. RIPK3 phosphorylates MLKL, a well-known functional substrate, in necrosomes. Following necroptosis, MLKL oligomerizes and translocates to the plasma membrane, where it causes the permeabilization of the necrotic plasma membrane and, ultimately, cell death, characterized by cell enlargement and a loss of cell and organelle integrity [346].

## 6. Translational Advances in Redox-Based Cancer Therapies

### 6.1. ROS-Modulating Drugs in Clinical Trials

The clinical effectiveness of therapeutic agents in cancer treatment is often constrained by major limitations, including the emergence of restricted therapeutic applicability, substantial toxicity, and drug resistance [347].

Elevated ROS levels and enhanced antioxidant defenses are frequently observed in drug-resistant cancer cells compared to their non-resistant or normal counterparts. This redox imbalance is increasingly recognized as a key contributor to therapeutic resistance. Understanding the specific roles of ROS in driving or sustaining drug resistance mechanisms is therefore essential for identifying strategies to re-sensitize refractory tumor cells. In light of the dual nature of ROS, therapeutic approaches involving antioxidants or agents that inhibit antioxidant systems are being explored as means to selectively modulate redox signaling in resistant cancers [348].

Multiple clinical trials have explored the therapeutic potential of redox-modulating agents in oncology, particularly in combination with established chemotherapeutics [349]. Among the agents that have reached advanced clinical testing, APR-246 has shown encouraging results in TP53-mutated myeloid malignancies when used alongside azacitidine, with several trials reaching Phases II and III. Similarly, agents targeting glutathione metabolism, such as buthionine sulfoximine, have been evaluated in early-phase studies for neuroblastoma and melanoma, often in combination with alkylating agents like melphalan. Drugs modulating redox-sensitive enzymes, such as glutamate-cysteine ligase (i.e., valproic acid and indomethacin), have progressed to Phase II trials in head and neck cancers, mesothelioma, and gastrointestinal malignancies, demonstrating potential in sensitizing tumors to platinum-based or anthracycline therapies. Inhibitors of mitochondrial function, including metformin and elesclomol, have also advanced to mid- and late-stage studies, particularly in breast, ovarian, and prostate cancers. Furthermore, mTOR pathway inhibitors (temsirolimus, everolimus) and redox-sensitive chaperone inhibitors (e.g., tanespimycin targeting HSP90) have entered Phase II and III investigations in solid tumors, such as breast, lung, and ovarian cancers. These trials reflect a growing clinical interest in leveraging redox biology not only to enhance cytotoxic efficacy but also to overcome resistance mechanisms associated with standard treatment regimens [350].

### 6.2. Synthetic Lethality Strategies Targeting Redox Vulnerabilities

Synthetic lethality (SL) describes a genetic interaction in which the concurrent disruption of two genes results in cell death, whereas the loss of either gene alone is tolerated. This principle is increasingly applied in oncology to exploit cancer-specific genetic alterations, allowing for selective targeting of tumor cells while minimizing toxicity to healthy tissue [351].

Recent insights into cancer metabolism have revealed that targeting redox homeostasis through SL offers a promising therapeutic strategy. Certain tumor types display an increased dependency on antioxidant systems, such as GSH, to buffer ROS. Inhibiting these systems exposes a redox vulnerability that can be exploited. For instance, the depletion of cysteine, essential for glutathione synthesis, synergizes with agents like sorafenib to trigger ferroptosis through oxidative stress. Additionally, AT-rich interaction domain 1A-deficient cancers show heightened sensitivity to glutamate-cysteine ligase catalytic subunit inhibition, leading to impaired glutathione synthesis and reactive oxygen species-induced apoptosis. Similarly, fumarate hydratase-mutant renal cancers exhibit elevated glycolytic activity and altered redox balance; inhibition of phosphogluconate dehydrogenase disrupts nicotinamide adenine dinucleotide phosphate production, inducing cytotoxic reactive oxygen species accumulation. These findings highlight that metabolic rewiring in tumors not only sustains their growth but also renders them vulnerable to oxidative stress when critical redox-supporting pathways are therapeutically targeted. Furthermore, simultaneous inhibition of glycolysis and mitochondrial oxidative phosphorylation in tumors with metabolic inflexibility amplifies redox imbalance and leads to selective cancer cell death. Thus, exploiting reactive oxygen species-linked metabolic dependencies through synthetic lethality provides a tailored approach to eliminate cancer cells while sparing normal tissue [352].

Several innovative anticancer agents, including inhibitors of the DNA damage response, protein arginine methyltransferase 5, methionine adenosyltransferase 2 alpha, glutaminase, and enhancer of zeste homologue 2, as well as degraders of SWI/SNF related matrix associated actin dependent regulator of chromatin subfamily A member 2, are currently being tested in early-phase clinical trials, either as single agents or in combination therapies [353,354].

To uncover redox vulnerabilities in cancer, researchers commonly employ CRISPR-Cas9-based negative selection screens. This approach allows systematic disruption of antioxidant-related genes to identify those whose loss increases cellular sensitivity to oxidative stress. By applying sublethal oxidative conditions, such as exposure to reactive oxygen species-inducing agents, this technique helps pinpoint key components of redox defense systems that may serve as therapeutic targets [355].

Relevant SL drugs that target redox vulnerabilities include sorafenib, which promotes ferroptosis by enhancing oxidative stress in tumor cells with depleted glutathione reserves. Inhibitors of glutamate-cysteine ligase catalytic subunit impair glutathione synthesis and selectively induce apoptosis in cancers deficient in AT-rich interaction domain 1A. STF-31 acts by inhibiting glucose transporter 1, leading to synthetic lethality in von Hippel–Lindau-deficient cells through glycolytic blockade. In fumarate hydratase-mutant tumors, inhibitors of phosphogluconate dehydrogenase disrupt nicotinamide adenine dinucleotide phosphate production, causing redox imbalance and cytotoxic accumulation of reactive oxygen species. Additionally, 2-deoxyglucose targets glycolysis and is synthetically lethal in β-catenin wild-type cancers, further underscoring the therapeutic value of exploiting metabolic redox dependencies [352].

### 6.3. Ferroptosis

Ferroptosis represents a regulated form of cell death that is dependent on iron and is marked by severe damage to cellular membranes due to the accumulation of lipid peroxides. Emerging evidence indicates that promoting ferroptosis can effectively suppress tumor progression in several cancer types. This strategy capitalizes on ferroptosis’s distinct mechanism, driven by iron-mediated lipid peroxidation, which provides a therapeutic avenue to bypass resistance to apoptosis, a frequent feature of malignant cells. Aberrant iron homeostasis and disrupted lipid profiles in tumors further enhance their vulnerability to ferroptosis induction [356].

Ferroptosis represents a novel therapeutic modality in cancer treatment, particularly relevant for tumors such as clear cell renal cell carcinoma and MYCN-amplified neuroblastoma, which exhibit intrinsic ferroptosis sensitivity. Initially identified as a mechanism of selective lethality in RAS-mutant cancer cells, subsequent evidence has demonstrated that cells with wild-type RAS can also undergo ferroptosis. Several compounds have been shown to induce this form of iron-dependent cell death through distinct pathways. For instance, altretamine suppresses glutathione peroxidase 4 activity, while withaferin A facilitates its degradation and promotes iron release from heme. Methotrexate impairs dihydrofolate reductase function, and statins disrupt the mevalonate pathway, both contributing to ferroptotic susceptibility. Additionally, APR-246 (eprenetapopt) depletes intracellular glutathione and inhibits cysteine desulfurase NFS1, and imetelstat increases polyunsaturated phospholipid abundance, enhancing lipid peroxidation. Collectively, these agents underscore the therapeutic potential of ferroptosis induction across genetically diverse tumor types [357,358].

Cells undergoing ferroptosis exhibit distinct morphological characteristics that differentiate this process from autophagy and apoptosis. Unlike apoptosis or autophagy, where the plasma membrane remains intact, ferroptosis involves increased membrane density followed by rupture, resulting in vesicle formation. Mitochondrial alterations are prominent in ferroptosis, including shrinkage and loss or reduction of cristae, while nuclear size typically remains unchanged except for chromatin condensation. Additionally, ferroptosis is characterized by excessive intracellular iron and reactive oxygen species accumulation. Thus, ferroptosis can be clearly distinguished from other regulated cell death pathways by its unique morphological and biochemical features. Regarding metabolic alterations, ferroptosis is associated with disruptions in iron metabolism, leading to intracellular iron overload that promotes free radical generation and oxidative damage. Lipid metabolic imbalances are also involved, as a higher proportion of monounsaturated fatty acids relative to polyunsaturated fatty acids in cancer cell membranes can inhibit lipid toxicity and ferroptosis. Furthermore, dysregulation of amino acid metabolism, particularly involving glutathione, plays a critical role in ferroptosis induction [359,360].

Ferroptosis functions as a natural tumor-suppressive process, contributing to the anticancer effects of multiple tumor suppressor genes. Key tumor suppressor genes, such as fumarate hydratase, the epigenetic regulator MLL4, Kelch-like ECH-associated protein 1, BRCA1-associated protein 1, and p53, have been demonstrated to promote tumor suppression partly through the induction of ferroptosis in cancer cells. Moreover, when ROS accumulate beyond a critical threshold, their pro-tumorigenic roles in cell proliferation and invasion are converted into antitumor responses by triggering regulated cell death pathways such as apoptosis, necroptosis, and notably ferroptosis [361].

Ferroptosis can be initiated to remove cancerous or precancerous cells through intrinsic signals from tumor suppressors, like p53, or via extrinsic cues, such as interferon-gamma produced by CD8-positive T cells. Despite its therapeutic promise in cancer elimination, ferroptosis is also involved in various pathological conditions, including ischemia–reperfusion injury and neurodegenerative diseases, which has sparked debate regarding its physiological roles in aging, tumor suppression, and infection control versus its detrimental effects. While ferroptosis induction holds considerable potential for targeting tumors, effectively directing this process specifically to malignant cells remains a significant challenge [358].

## 7. Interplay of ROS, Inflammation, and Cancer

According to Virchow’s hypothesis, inflammation is linked to subsequent cancer development. Over time, numerous researchers confirmed Virchow’s theory and discovered that chronic inflammation controls the progression of cancer depending on the number of inflammation-related factors, such as inflammatory cytokines and chemokines, either by inducing an antitumor response or by promoting malignant cell transformation [362]. The type, location, and levels of inflammation-modulating factors, such as activator protein 1 (AP-1), catenin/Wnt (wingless related integration site), NF-B, PPAR-gamma, HIF-1 alpha (hypoxia-inducible factor-1 alpha), p53, inflammatory cytokines, chemokines, and growth factors, are all influenced by ROS; as a consequence, ROS play an essential part in establishing the relationship between chronic inflammation and cancer. iNOS, myeloperoxidase (MPO), NADPH oxidase, and xanthine oxidase (XO), as well as upregulated cyclooxygenase 2 (COX2) and lipoxygenase (LOX), are just a few of the oxidant-producing enzymes that the inflammatory cells in the tumor microenvironment activate [363,364,365].

Increased ROS damages the cell’s proteins, lipids, DNA, RNA, and mitochondria, resulting in over-mutation, disrupted signaling pathways, apoptosis inactivation, and further overproduction of ROS that eventually stimulates inflammatory cytokines, chemokines, and molecules that drive inflammation [363,365,366,367,368]. In solid tumors, a poor microvascular network and high interstitial pressure create a hypoxic condition that stimulates HIF expression. HIFs govern mitochondrial metabolism and the emergence of ROS; in turn, mitochondrial ROS control the expression of HIFs. Active NF-κB is thought to play a significant role in the development of malignancies that are resistant to therapies (i.e., gamma radiation and many chemotherapeutic drugs), in addition to creating chronic inflammation. Bcl-2, Bcl-xL, Akt, cyclin D1, COX-2, survivin, and XIAP (X-linked inhibitor of apoptosis) have all been implicated in its occurrence through transcriptional upregulation [369,370,371,372,373,374]. It ought to be kept in mind that in the triangle formed by ROS, inflammation, and cancer, focusing on ROS may be a very promising strategy to reduce chronic inflammation linked to cancer and other manifestations of cancer development.

## 8. Conclusions and Prospects

The present narrative review offers a comprehensive analysis of ROS in cancer biology, distinguished by its integration of biochemical mechanisms, redox-sensitive signaling pathways, ROS detection and quantification tools, and the dual roles of ROS in both tumor promotion and suppression. It goes beyond classical models by incorporating multiple translational, biochemical, and therapeutic items, from ROS-modulating drugs in clinical trials and synthetic lethality strategies to ferroptosis as a redox-driven cell death pathway, effectively bridging fundamental redox biology with emerging therapeutic approaches.

Despite these advances, several key gaps remain. The context-specific effects of ROS, dictated by their levels, localization, and duration, require further elucidation, especially in relation to immune evasion, metabolic reprogramming, and treatment resistance. Functional heterogeneity among ROS-generating enzymes across tumor microenvironments is also insufficiently understood. Future research should employ genetically encoded biosensors for real-time, in vivo ROS mapping to better exploit its double-edged properties in precision oncology, redox proteomics to identify therapeutic post-translational modifications, and high-throughput CRISPR screens to uncover redox-based vulnerabilities. Clarifying the redox thresholds that tip the balance from signaling to cytotoxicity may enable more precise therapeutic targeting.

Ultimately, integrating redox biology with immunotherapy, metabolic modulation, and precision oncology could reshape cancer treatment. A deeper understanding of ROS dynamics will be crucial for harnessing their antitumor potential while minimizing pro-tumorigenic consequences.

## Figures and Tables

**Table 1:** Some of the factors that may trigger cancer by activating ROS-mediated cell signaling pathways.

Stimulus/Receptor	Model/Cell Line/Cancer Type	Signaling Pathway/ Factor Involved	Ref
**Angiogenesis**			
Human papillomavirus E7 oncoprotein	Cervical cancer cells	HIF-1α/VEGF/ERK1/2	[39]
Arsenite	DU145 human prostate#break# carcinoma cells	PI3K/Akt	[40]
27-hydroxycholesterol	Human breast cancer cells	STAT-3/VEGF	[41]
Angiopoietin-1 (Ang1)	Human umbilical vein endothelial cells	p44/42/MAPK	[42]
Ubiquitin C-terminal hydrolase-L1 (UCH-L1)	B16F10 cells	VEGF	[43]
EGF/EGFR	Human ovarian cancer cells	AKT/p70S6K1	[44]
Centchroman	Human breast cancer cells	AKT/ERK	[45]
Arsenic	Human lung epithelial BEAS-2B cells	miR-199a-5p/HIF-1α/COX-2	[46]
NADPH oxidase subunit p22phox	Human prostate cancer	AKT/ERK/HIF-1/VEGF	[47]
**Carcinogenesis**			
Farnesoid X Receptor	Liver cell	JNK	[48]
1,2-dimethylhydrazine (DMH)	Colon cell	PI3-K/Akt/GSK-3β/PTEN	[49]
Hepatitis C virus (HCV Core, E2, NS3, NS5A)	HUH7 cells	MAPK/ERK	[50]
Epstein–Barr virus (EBV) nuclear antigen (EBNA)-1	Lymphoma	NF-κB/MAPK	[51]
*Helicobacter pylori*	AGS human gastric cancer cells	STAT3/IL-6 and IL-6R	[52]
Environmental and dietary carcinogens	Breast cancer	RAS-ERK	[53]
Particulate matter	Lung cancer	p16 promoter/JNK inhibitor	[54]
K- rasV12	Non-transformed peripheral mouse lung epithelial cells (E10 line)	COX-2	[55]
**Carcinogenesis/Metastasis**			
Human T-cell lymphotropic virus type 1 (HTLV-1)	CD4+ T lymphocytes	TBK1-mediated phosphorylation of IRF3/IRF7	[56]
Invasion/metastasis			
Slingshot-1L (SSH-1L)	HeLa cells	Cofilin	[57]
Neuregulin (NRG)	HaCaT keratinocytes	SSH-1L/cofilin	[58]
Inhibition of autophagy	Gastric cancer	NF-κB	[59]
ITGB3	Colorectal cancer (CRC)	MAPK/AKT	[60]
Capsaicin	SW480 cells	Akt/mTOR and STAT-3	[61]
Ionizing radiation (IR)	Epithelial cells	EGFR/PI3K/Akt/MAPK	[62]
Benzo[a]pyrene	Breast cancer cells	ROS-ERK-MMP9	[63]
**Metastasis**			
Fork head box M1 (FoxM1) of Hepatitis C virus	Liver cells	ERK/CREB	[64]
**Tumorigenesis**			
As(III) or Cr(VI)	Mouse colitis-linked colorectal cancer model	Wnt/β-catenin	[65]
TRPC3	Gastric cancer	CNB2/GSK3β/NFATc2	[66]
*S. japonicum* SjE16.7 Protein	Colorectal cancer	NF-κB/IL-6 and TNF-α.	[67]
**Tumorigenesis and Angiogenesis**			
Cadmium (CdCl2)	Lung epithelial cell	ERK/AKT	[68]
**Tumorigenesis and Metastasis**			
Cholesterol (LDL)	Colorectal adenoma and colorectal cancer	MAPK	[69]
**Tumorigenesis/Angiogenesis**			
Kaposi’s sarcoma herpesvirus (KSHV)	mECK36	vGPCR/VEGF	[70]
**Tumorigenesis/Angiogenesis/Metastasis**			
Chronic alcohol	Liver cell	MAPK/RAS/Rb/TGFβ/p53/PTEN/ECM	[71]

Ref, reference; MAPK, mitogen-activated protein kinase; ERK/AKT, extracellular signal-regulated kinase/protein kinase B; CNB2/GSK3β/NFATc2, calcineurin B homologous protein 2/glycogen synthase kinase 3 beta/nuclear factor of activated T cells cytoplasmic 2; JNK, c-Jun N-terminal kinase; PI3-K/Akt/GSK-3β/PTEN, phosphoinositide 3-kinase/protein kinase B/glycogen synthase kinase 3 beta/phosphatase and tensin homolog; MAPK/ERK, mitogen-activated protein kinase/extracellular signal-regulated kinase; HIF-1α/VEGF/ERK1/2, hypoxia-inducible factor 1-alpha/vascular endothelial growth factor/extracellular signal-regulated kinase 1/2; ERK/CREB, extracellular signal-regulated kinase/cAMP response element-binding protein; NF-κB/MAPK, nuclear factor kappa-light-chain-enhancer of activated B cells/mitogen-activated protein kinase; TBK1-mediated phosphorylation of IRF3/IRF7, TANK-binding kinase 1/interferon regulatory factor 3/interferon regulatory factor 7; vGPCR/VEGF, viral G protein-coupled receptor/vascular endothelial growth factor; STAT3/IL-6 and IL-6R, signal transducer and activator of transcription 3/interleukin-6 and interleukin-6 receptor; NF-κB/IL-6 and TNF-α, nuclear factor kappa-light-chain-enhancer of activated B cells/interleukin-6 and tumor necrosis factor-alpha; RAS-ERK, renin–angiotensin system/extracellular signal-regulated kinase; p16 promoter/JNK inhibitor, cyclin-dependent kinase inhibitor 2A promoter/c-Jun N-terminal kinase inhibitor; MAPK/RAS/Rb/TGFβ/p53/PTEN/ECM, mitogen-activated protein kinase/renin–angiotensin system/retinoblastoma/transforming growth factor-beta/p53/phosphatase and tensin homolog/extracellular matrix; COX-2, cy-clooxygenase-2; PI3K/Akt, phosphoinositide 3-kinase/protein kinase B; Cofilin, cofilin; STAT-3/VEGF, signal transducer and activator of transcription 3/vascular endothelial growth factor; p44/42/MAPK, p44/42 mitogen-activated protein kinase; VEGF, vascular endothelial growth factor; SSH-1L/cofilin, slingshot protein phosphatase 1L/cofilin; AKT/p70S6K1, protein kinase B/ribosomal protein S6 kinase beta-1; AKT/ERK, protein kinase B/extracellular signal-regulated kinase; ERK1/2, extracellular signal-regulated kinase 1/2; miR-199a-5p/HIF-1α/COX-2, microRNA-199a-5p/hypoxia-inducible factor 1-alpha/cyclooxygenase-2; AKT/ERK/HIF-1/VEGF, protein kinase B/extracellular signal-regulated kinase/hypoxia-inducible factor 1/vascular endothelial growth factor; NF-κB, nuclear factor kappa-light-chain-enhancer of activated B cells; MAPK/AKT, mitogen-activated protein kinase/protein kinase B; Akt/mTOR and STAT-3, protein kinase B/mammalian target of rapamycin/signal transducer and activator of transcription 3; EGFR/PI3K/Akt/MAPK, epidermal growth factor receptor/phosphoinositide 3-kinase/protein kinase B/mitogen-activated protein kinase; ROS-ERK-MMP9, reactive oxygen species/extracellular signal-regulated kinase/matrix metalloproteinase-9; ITGB3, integrin beta-3; EGF/EGFR, epidermal growth factor/epidermal growth factor receptor; TRPC3, transient receptor potential cation channel subfamily C member 3; LDL, low-density lipoprotein; As(III), arsenite; Cr(VI), hexavalent chromium.

**Table 2:** Several pharmacological agents leading to cell death via ROS-mediated cell signaling pathways classified by outcome.

Stimulant/Drug	Model System/Cell Line/Cancer Type	Signaling Pathway/ Factor Involved	Ref.
**Apoptosis**			
Isoobtusilactone A	Human breast cancer cells	ASK1	[223]
Hyperoside (quercetin 3-o-β-d-galactopyranoside)	Breast cancer cells	NF-κB	[224]
Schisantherin A	Human gastric cancer cells	JNK	[225]
Resveratrol	HT-29 cells	AMPK	[226]
5-fluorouracil and genistein	HT-29 colon cancer cells	AMPK/COX-2	[227]
Rhein	Liver cancer cells	JNK/Jun/caspase-3	[228]
Delicaflavone	Colorectal cancer cells	PI3K/AKT/mTOR and Ras/MEK/Erk	[229]
Eupatilin	Human renal cancer cells	MAPK and PI3K/AKT	[230]
Sulforaphane	Human urinary bladder cancer T24	Nrf2	[231]
Emodin	Human lung adenocarcinoma cells	ERK and AKT	[232]
Carnosic acid	Cervical cancer cells	JNK	[233]
Tetramethyl pyrazine	Gastric cancer cells	AMPK	[234]
Zeaxanthin	Human gastric cancer cells	MAPK and AKT	[235]
Atmospheric gas plasmas (AGPs)	Melanoma cancer cells	TNF-ASK1	[236]
7-O-Geranylquercetin	Gastric cancer cell lines SGC-7901 and MGC-803	MAPK	[237]
CDK5RAP1	Human breast cancer cells	JNK	[238]
Isoorientin	HepG2 cancer cells	PI3K/Akt	[239]
Benzimidazole acridine derivative (8m)	Human colon cancer cell lines SW480 and HCT116	JNK1	[240]
Echinatin	Colorectal cancer cells HCT116 and HCT8 cell	JNK/p38 MAPK	[241]
Emodin	Colorectal cancer cells SW480 and SW620	p38/p53/Puma	[242]
Sulforaphane	p53-deficient SW480 cells	MAPK	[243]
*E. scaber* ethanol extract	Human colorectal carcinoma cells HCT116	p53	[244]
Shikonin and 4-hydroxytamoxifen	Human breast cancer cells	PI3K/AKT/Caspase 9	[245]
Selenadiazole	Bladder cancer cells	AKT and MAPKs	[246]
Licochalcone A	Human gastric cancer BGC-823	MAPKs and PI3K/AKT	[247]
Alpha-mangostin	Cervical cancer HeLa and SiHa cells	ASK1/p38	[248]
Hypocrellin A	Human lung adenocarcinoma A549 cells	Cytochrome c	[249]
EGCG [(−) epigallocatechin-3-gallate]	Colon cancer cells HT-29	AMPK	[250]
Naringenin	Prostate cancer cells PC3 and LNCaP	PI3K/AKT and MAPK	[251]
Curcumin	Human lung adenocarcinoma A549 cells	MAPK	[252]
Quinalizarin	Human Breast cancer cells	MAPK, STAT3 and NF-κB	[253]
Verrucarin A	Human Breast cancer cells MDA-MB-231	EGFR/MAPK/Akt	[254]
Ginsenosides (GRg3 and GRh2)	Human hepatocellular carcinoma cells Hep3B	Cytochrome c	[255]
Fucoidan	Human bladder cancer cells 5637	PI3K/Akt	[256]
BQ and OQ	Gastric cancer cell lines	MAPK/Akt/STAT3	[257]
Melatonin	Gallbladder cancer cells NOZ and GBC-SD cells	PI3K/Akt/mTOR	[258]
Isoorientin	Human hepatoblastoma cancer cells HepG2	p53, PI3K/Akt, JNK, and p38	[259]
Camel Milk	Human hepatoma HepG2 and breast cancer MCF7	Caspase-3 mRNA	[260]
Benzyl isothiocyanate	Human melanoma cells A375.S2	Cytochrome c	[261]
Broussochalcone A	Human renal cancer cells A498 and ACHN	FOXO3	[262]
Britannin, a sesquiterpene lactone	Human breast cancer cells MCF-7 and MDA-MB-468	Cytochrome c	[263]
Phyllanthin	Leukemic cancer cells MOLT-4	AKT/JNK	[264]
Erlotinib	Lung cancer cell lines A549	JNK	[265]
SL4 (chalcone)	Hep3B and MDA-MB-435	MAPK	[266]
Voacamine	MCF-7	PI3K/Akt/mTOR	[267]
**Apoptosis/Autophagy**			
Bruceine D	Lung cancer cells	MAPK	[268]
Physagulide P	MDA-MB-231 and MDA-MB-468 cells	JNK	[269]
Momordin Ic	HepG2 cells	PI3K/Akt and MAPK	[270]
Celastrol	Human osteosarcoma cells	JNK	[271]
Costunolide	Renal cell carcinoma 786-O, A-498, ANCH, and 769-P	JNK/MAPK	[272]
Cedrol	Human lung carcinoma cells A549	P13K/Akt	[273]
Propranolol	Human ovarian cancer cell lines SKOV-3 and A2780	JNK	[274]
Honokiol	Human osteosarcoma cells	ERK1/2	[275]
Curcin C	Osteosarcoma cell lines U2OS	JNK	[276]
Juglanin	Human breast cancer cells	JNK	[277]
PPM-18	Human bladder cancer cell line T24	AMPK	[278]
Metformin	Human osteosarcoma cells U2OS and 143B	JNK	[279]
Graveoline	Skin melanoma A375 cells	PI3K	[280]
Triptolide	Glioma cell U251, U87-MG	JNK/Akt/mTOR	[281]
Erianin	Osteosarcoma cells 143B, MG63.2	JNK	[282]
Anethole	Oral cancer cells Ca9-22	NF-κB/MAPK/p53	[283]
Arsenic sulfide	Osteosarcoma cell lines 143B, MG-63, HOS and U2OS	JNK/Akt/mTOR	[284]
Parthenolide	Cervical cancer HeLa cells	PI3K/Akt	[285]
1,3-dibutyl-2-thiooxo-imidazolidine-4,5-dione	Colorectal carcinoma cells HCT116	ERK/JNK	[286]
6-Methoxydihydrosanguinarine	Breast cancer cells MCF-7	PI3K/AKT/mTOR	[287]
**Autophagy**			
α-hederin	Colorectal cancer cells HCT116 and HCT8 cell	AMPK/mTOR	[288]
m-THPC	Human colorectal cancer cells ATG5 or ATG7	JNK	[289]
Luteoloside	NSCLC (A549 and H292) cells	AKT/mTOR/p70S6K	[290]
Diosgenin	Chronic myeloid leukemia cells	mTOR	[291]
Cryptotanshinone	Human colon cancer cell line SW620 Ad300	p38/MAPK/NF-κB	[292]
Piperlongumine	Gallbladder cancer cells OCUG-1	ErK	[293]
Mitofusin2	Pancreatic cancer cell Aspc-1	PI3K/Akt/mTOR	[294]
SZC017 Oleanolic acid derivative	Human lung cancer cells A549	Akt/JAK2/STAT3	[295]
Designed siRNAs for cyclinB1 mRNA	Nasopharyngeal carcinoma cells CNE-1 and CNE-2	AMPK-ULK1	[296]
Eriocalyxin B	Breast cancer cell lines MCF-7 and MDA-MB-231	Akt/mTOR/p70S6K	[190]
Chrysin	Human endometrioid adenocarcinoma cell line HEC-1A	Akt/mTOR	[297]
*Dendrobium officinale* polysaccharide	Colon cancer cell line CT26	AMPK	[298]
**Necroptosis**			
2-methoxy-6-acetyl-7-methyljuglone	Colon cancer cells HCT116 and HT29	JNK	[299]
Ergothioneine	Colon cancer cells HT-29	SIRT3/MLKL	[300]
Dimethyl fumarate	Colon cancer cells CT26	MAPKs and PI3K/AKT	[301]
Tanshinol A	Lung cancer cells	MLKL	[302]
Goniothalamin	Human invasive breast cancer cells MDA-MB-231	EGFR/FAK/Src	[303]
Gallic Acid	Primary hepatic cells (HCs) and hepatic stellate cells (HSCs)	TNF–α	[304]
Givinostat/Sorafenib	Hodgkin’s lymphoma cell lines HDLM-2 and L-540 HL	BIM	[305]

BQ, 2-(butane-1-sulfinyl)-1,4-naphthoquinone; OQ, 2-(octane-1-sulfinyl)-1,4-naphthoquinone; ASK1, apoptosis signal-regulating kinase 1; MAPK, mitogen-activated protein kinase; NF-κB, nuclear factor kappa-light-chain-enhancer of activated B cells; JNK, c-Jun N-terminal kinase; AMPK, AMP-activated protein kinase; COX-2, cyclooxygenase-2; Jun, transcription factor AP-1 subunit; caspase-3, caspase-3; PI3K, phosphoinositide 3-kinase; AKT, protein kinase B; mTOR, mammalian target of rapamycin; Ras, renin–angiotensin system; MEK, mitogen-activated protein kinase kinase; Erk, extracellular signal-regulated kinase; Nrf2, nuclear factor erythroid 2-related factor 2; p38, p38 MAP kinase; p53, tumor protein p53; Puma, p53 upregulated modulator of apoptosis; Caspase-9, caspase-9; STAT3, signal transducer and activator of transcription 3; EGFR, epidermal growth factor receptor; FAK, focal adhesion kinase; Src, proto-oncogene tyrosine-protein kinase Src; TNF-α, tumor necrosis factor-alpha; BIM, Bcl-2-like protein 11; FOXO3, fork head box O3; Cytochrome c, cytochrome c; SIRT3, sirtuin-3; MLKL, mixed-lineage kinase domain-like; EGFR, epidermal growth factor receptor; TNF–α, tumor necrosis factor-alpha.

## Data Availability

No new data were created or analyzed in this study. Data sharing is not applicable to this article.

## Abbreviations

The following abbreviations are used in this manuscript:

ROS	Reactive oxygen species
NADPH	Nicotinamide adenine dinucleotide phosphate (reduced form)
NADP⁺	Nicotinamide adenine dinucleotide phosphate (oxidized form)
GR	Glutathione reductase
GSH	Reduced glutathione
GSSG	Oxidized glutathione
GPx/GPX	Glutathione peroxidase
TRX	Thioredoxin
TRX red	Reduced thioredoxin
TRX ox	Oxidized thioredoxin
PRDX/Prx	Peroxiredoxin
SOD	Superoxide dismutase
HO•	Hydroxyl radical
O_2_^2−^/O_2_•^−^	Superoxide ion/Superoxide anion radical
H_2_O_2_	Hydrogen peroxide
MAPK	Mitogen-activated protein kinase
ERK	Extracellular signal-regulated kinase
AKT	Protein kinase B
JNK	c-Jun N-terminal kinase
PI3K	Phosphoinositide 3-kinase
mTOR	Mammalian target of rapamycin
PTEN	Phosphatase and tensin homolog
NF-κB	Nuclear factor kappa-light-chain-enhancer of activated B cells
VEGF	Vascular endothelial growth factor
VEGFR	Vascular endothelial growth factor receptor
MMPs/MMP-9	Matrix metalloproteinases/Matrix metalloproteinase-9
COX-2	Cyclooxygenase-2
STAT1/3	Signal transducer and activator of transcription 1/3
STAT-3	Signal transducer and activator of transcription 3
IL-6/IL-6R	Interleukin-6/Interleukin-6 receptor
IL-1β	Interleukin-1 beta
TNF-α	Tumor necrosis factor-alpha
TNFR	Tumor necrosis factor receptor
TLR4	Toll-like receptor 4
TRAF2/TRAF6	TNF receptor-associated factor 2/6
EGFR	Epidermal growth factor receptor
EGF	Epidermal growth factor
PDGF	Platelet-derived growth factor
TKR	Tyrosine kinase receptor
NOX/NOX2	NADPH oxidase/NADPH oxidase 2
ER	Endoplasmic reticulum
p53	Tumor protein p53
RAS	Renin–angiotensin system
RAF	Rapidly accelerated fibrosarcoma
MEK	Mitogen-activated protein kinase kinase
MKK	MAP kinase kinase
ASK	Apoptosis signal-regulating kinase
IKK-α/IKK-β	IκB kinase alpha/beta
NEMO	NF-κB essential modulator
IκB	Inhibitor of kappa B
p50/p65	NF-κB subunits 1/3
NFAT	Nuclear factor of activated T cells
CREB	cAMP response element-binding protein
HO-1	Heme oxygenase-1
GPX1	Glutathione peroxidase 1
SHP-2	Src homology region 2-containing phosphatase-2
PKAc	Protein kinase A catalytic subunit
Keap1	Kelch-like ECH-associated protein 1
NRF2	Nuclear factor erythroid 2-related factor 2
Cul3	Cullin-3
HIF-1α/HIF-1β	Hypoxia-inducible factor 1-alpha/beta
PHDs	Prolyl hydroxylases
P-VHL	von Hippel–Lindau tumor suppressor protein
c-Myc	Cellular Myc oncogene
Elk1	Ets-like protein 1
Co-filin	Actin-binding protein cofilin
SSH-1L	Slingshot protein phosphatase 1L
p44/42 MAPK	p44/42 mitogen-activated protein kinase
xCT	Cystine/glutamate antiporter
GSR	Glutathione reductase
G6PD	Glucose-6-phosphate dehydrogenase
GCL	Glutamate-cysteine ligase
TXNRD1	Thioredoxin reductase 1
PRDX	Peroxiredoxin
CXCR4	C-X-C chemokine receptor type 4
Bcl-2/Bcl-XL	B-cell lymphoma 2/B-cell lymphoma-extra-large
LPS	Lipopolysaccharide
miR-199a-5p	MicroRNA-199a-5p
As(III)	Arsenite
Cr(VI)	Hexavalent chromium
ITGB3	Integrin beta-3
TRPC3	Transient receptor potential cation channel subfamily C member 3
LDL	Low-density lipoprotein
ECM	Extracellular matrix
TGF-β	Transforming growth factor-beta
Cyclin D1	Cell cycle regulatory protein D1
TPA	12-O-tetradecanoylphorbol-13-acetate
CYLD	Cylindromatosis tumor suppressor protein
EMT	Epithelial–mesenchymal transition
SERS	Surface-Enhanced Raman Spectroscopy
SUMO	Small ubiquitin-like modifier
PTM	Post-translational modification
SL	Synthetic lethality
